# Genetic polymorphisms associated with susceptibility to COVID-19 disease and severity: A systematic review and meta-analysis

**DOI:** 10.1371/journal.pone.0270627

**Published:** 2022-07-06

**Authors:** Cristine Dieter, Letícia de Almeida Brondani, Cristiane Bauermann Leitão, Fernando Gerchman, Natália Emerim Lemos, Daisy Crispim

**Affiliations:** 1 Endocrine and Metabolism Division, Hospital de Clínicas de Porto Alegre, Porto Alegre, Rio Grande do Sul, Brazil; 2 Postgraduate Program in Medical Sciences: Endocrinology, Faculdade de Medicina, Universidade Federal do Rio Grande do Sul, Porto Alegre, Rio Grande do Sul, Brazil; Universita degli Studi di Roma Tor Vergata, ITALY

## Abstract

Although advanced age and presence of comorbidities significantly impact the variation observed in the clinical symptoms of COVID-19, it has been suggested that genetic variants may also be involved in the disease. Thus, the aim of this study was to perform a systematic review with meta-analysis of the literature to identify genetic polymorphisms that are likely to contribute to COVID-19 pathogenesis. Pubmed, Embase and GWAS Catalog repositories were systematically searched to retrieve articles that investigated associations between polymorphisms and COVID-19. For polymorphisms analyzed in 3 or more studies, pooled OR with 95% CI were calculated using random or fixed effect models in the Stata Software. Sixty-four eligible articles were included in this review. In total, 8 polymorphisms in 7 candidate genes and 74 alleles of the *HLA* loci were analyzed in 3 or more studies. The HLA-A*30 and *CCR5* rs333Del alleles were associated with protection against COVID-19 infection, while the *APOE* rs429358C allele was associated with risk for this disease. Regarding COVID-19 severity, the HLA-A*33, *ACE1* Ins, and *TMPRSS2* rs12329760T alleles were associated with protection against severe forms, while the HLA-B*38, HLA-C*6, and *ApoE* rs429358C alleles were associated with risk for severe forms of COVID-19. In conclusion, polymorphisms in the *ApoE*, *ACE1*, *TMPRSS2*, *CCR5*, and *HLA* loci appear to be involved in the susceptibility to and/or severity of COVID-19.

## Introduction

Coronavirus disease 2019 (COVID-19), caused by the severe acute respiratory syndrome coronavirus 2 (SARS-CoV-2), was identified in China near the end of 2019, and progressed to a pandemic condition in March 2020, resulting in a major public health problem worldwide due to its social and economic burdens [1]. As of February 1, 2022, COVID-19 affected more than 370 million people, and caused more than 5,658,702 deaths (https://www.who.int/publications/m/item/weekly-operational-update-on-covid-19—1-february-2022).

Clinical manifestations of COVID-19 vary from an asymptomatic infection, dry cough, sore throat, fever, shortness of breath, fatigue, muscle pain, headache, loss of taste or smell, vomiting, diarrhea, to acute respiratory distress syndrome. Approximately 15% of patients develop the severe form, which can progress to pneumonia, respiratory failure, kidney injury, multiorgan dysfunction, and death [2, 3]. The variation in symptoms and severity of COVID-19 is partially explained by known risk factors, including advanced age, male gender, and presence of comorbidities, such as diabetes, obesity, hypertension, and heart disease [4, 5]. However, severe outcomes have also been observed in young and healthy patients, suggesting that other risk factors, such as genetic predisposition, may increase the risk to and/or severity of this disease [6–8].

It is well known that host genetic polymorphisms play a key role in the susceptibility or resistance to different viral infections [9, 10]. Taking into account the main role of host genes in the entry and replication of SARS-CoV-2 in cells and in mounting the immune response, it seems that a combination of multiple genes might be involved in COVID-19 pathogenesis [9]. Accordingly, to date, numerous studies have been conducted on the association between genetic polymorphisms and COVID-19 [6, 7, 9–11]. Some studies have indicated that polymorphisms in genes related to innate and adaptive immune response [toll-like receptors (*TLRs*), human leukocyte antigen (*HLA*) class I and II, and cytokines/chemokines] and in genes involved in viral binding and entry into host cells (angiotensin converting enzyme-2 –*ACE2*, and transmembrane serine protease–*TMPRSS*) are associated with COVID-19 development and/or severity [6–8, 12]. However, it is still unclear which and to what degree specific polymorphisms contribute to the susceptibility for this disease [6].

Thus, aiming to identify the genetic factors that may influence COVID-19 susceptibility and severity, we conducted a comprehensive and updated systematic review of the literature on the subject followed by meta-analyses of those polymorphisms analyzed in three or more studies. Even though few systematic reviews have been published regarding the association between polymorphisms in different genes and COVID-19 [6, 7, 10, 12].

## Materials and methods

### Literature search strategy and eligibility criteria

This comprehensive and updated systematic review was performed and written according to the Preferred Reporting Items for Systematic Reviews and Meta-Analyses (PRISMA), Meta-analysis of Observational Studies in Epidemiology (MOOSE) statements and guideline for Systematic Reviews of Genetic Association Studies [13–15], and it was registered at PROSPERO (http://www.crd.york.ac.uk/PROSPERO) under the CRD42021248091 number. We performed a search at PubMed and Embase repositories for all English, Portuguese, and Spanish language original articles that analyzed potential associations between genetic polymorphisms and susceptibility/severity for COVID-19, up to July, 2021. For this, the following MeSH terms were used: (SARS-CoV-2 OR COVID-19 OR severe acute respiratory syndrome OR SARS virus) AND (polymorphism, genetic OR polymorphism, single nucleotide OR polymorphism, single-stranded conformational OR polymorphism, restriction fragment length OR DNA copy number variations OR amplified fragment length polymorphism analysis OR mutation OR mutation rate OR INDEL mutation OR mutation, missense OR point mutation OR frameshift mutation OR codon, nonsense). In addition, studies of interest were also searched in the GWAS Catalog (https://www.ebi.ac.uk/gwas).

Two independent investigators (C.D and L.A.B) screened and evaluated the eligibility of each study retrieved from the online repositories by reviewing titles and abstracts. When abstracts did not provide adequate information, the full texts of the extracted articles were also reviewed, as previously reported by our group [16, 17]. Discrepancies between the two investigators were settled by debate between them and, when necessary, a third reviewer (D.C.) was consulted. All observational human studies that compared frequencies of at least one polymorphism between patients with and without COVID-19 or between COVID-19 patients with different degrees of severity were included in this systematic review. Moreover, reference lists coming from the articles fulfilling our eligibility criteria were manually searched to identify other potentially relevant citations.

The exclusion criteria were: 1) articles without enough data to estimate an OR with 95% CI; 2) duplicated studies (in this case, the most complete study was chosen for inclusion); and 3) non-human studies.

### Data extraction and quality evaluation

Necessary information from each study was individually extracted by C.D. and L.A.B. using a standardized form [16, 17]. Agreement was pursued in all evaluated items of this form; however, when an agreement could not be reached, divergences in data extraction were solved by referring to the original article or by consulting another investigator (D.C.). Data retrieved from each study were as follows: 1) characteristics of the studies and samples (including publication year, name of first author, number of subjects in each analyzed group, mean age, gender, country, and ethnicity); and 2) data of the polymorphisms of interest [including their identification, allele/genotype frequencies, and OR (95% CI)]. When data were not available in the article, the authors were contacted by email for the necessary information, but only part of them answered.

The Clark-Baudouin Score (CBS) was used to evaluate the quality of the included studies [18]. This score applies pre-defined criteria to assess each publication, highlighting quality issues in the conduction of studies and interpretation of results. Using a 10-point scoring sheet, investigators can evaluate sections of the articles related to reproducibility, selection of subjects, statistical analyses, and genotyping methods.

### Statistical analyses for meta-analysis

Those polymorphisms analyzed in three or more studies were submitted to meta-analyses using the Stata 15.0 software (StataCorp, College Station, TX, USA). Goodness-of-fitness χ^2^ tests were used to evaluate whether genotype frequencies were in conformity with the Hardy-Weinberg Equilibrium (HWE) in the control groups. Associations between individual polymorphisms and COVID-19 susceptibility and/or severity were analyzed using OR (95% CI) calculations for the allele contrast, dominant, recessive, and additive inheritance models, categorized as suggested by a previous publication [19]. For the *HLA* allelic analysis, frequency was calculated as the number of cases or controls harbouring at least one positive event (one allele type) divided by the total number of chromosomes included in each of the corresponding groups [20]. Inter-studies heterogeneity was tested using χ^2^-based Cochran’s Q statistic, while inconsistency was quantified with the I^2^ metric [21, 22]. When P < 0.10 (Q statistic) and/or I^2^ > 50%, heterogeneity was considered statistically relevant. In this case, the DerSimonian and Laird random effect model (REM) was used to calculate OR (95% CI) for each study and for the pooled effect. In the lack of significant inter-studies heterogeneity, the fixed effect model (FEM) was used for this calculation.

## Results

### Literature search

**Fig 1:**
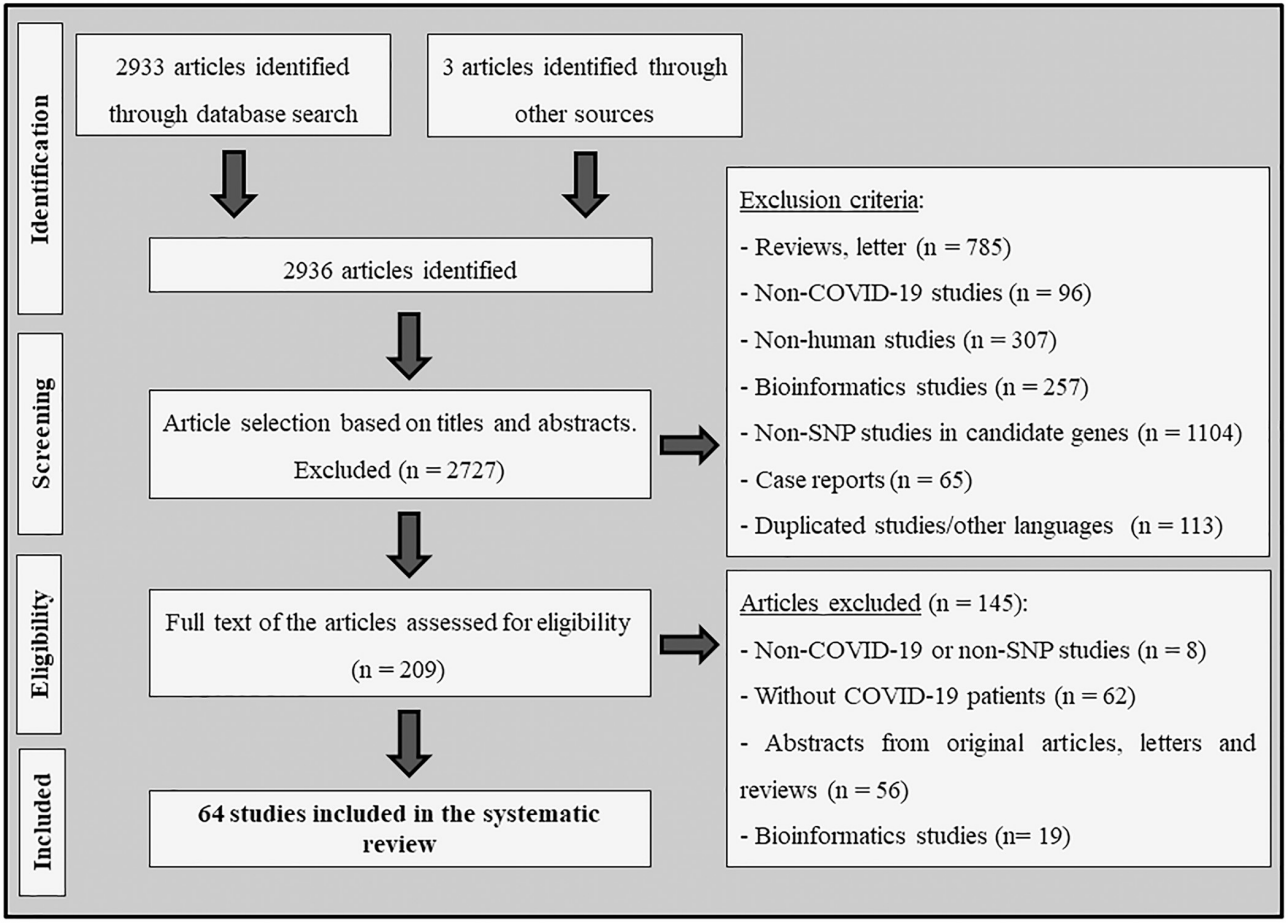
Flowchart illustrating the search strategy used to identify association studies between genetic polymorphisms and COVID-19 disease.

**Fig 1** shows the flow diagram illustrating the strategy used to identify and select studies for inclusion in our systematic review and meta-analyses. A total of 2936 articles were retrieved after searching PubMed, Embase, and GWAS Catalog resources, and 2727 of them were excluded during the review of titles and abstracts due to disagreements with our defined eligibility criteria. Two hundred and nine articles remained to be full text evaluation. Nevertheless, after carefully analyzing the full texts, another 145 studies were excluded, and a total of 64 articles were included in this systematic review (**Table 1** and **Fig 1**). Among them, 30 studies, where the same SNP was evaluated in at least 3 articles and frequency data was available, were included in the meta-analyses.

**Table 1:** Characteristics of studies included in the systematic review.

Reference	Population	Sample (case/control)	Gene	Results
Agwa *et al*., 2021 [23]	Egyptian	141 cases / 100 controls	*INFλ*, *TLL1*, *DDR1*	Disease susceptibility: The *IFN-λ* rs12979860 C/C, *TLL1* rs17047200 A/A and the *DDR1* rs4618569 A/A genotypes were associated with COVID-19 (P = 0.011, P = 0.012, and P = 0.026, respectively). Severity: The *DDR1* rs4618569 A/G was associated with COVID-19 severity (P = 0.007).
Alghamdi *et al*., 2021 [24]	Saudi	880 cases	*IFITM3*	Disease susceptibility: The rs12252 G allele was associated with risk for hospital admission (OR = 1.65, 95% CI 1.01–2.70, P = 0.04). Severity: The rs12252 G allele conferred risk for mortality (OR = 2.2, 95% CI 1.16–4.20, P = 0.01).
Amodio *et al*., 2020 [25]	Italian	381 cases	*IFNL3*, *IFNL4*	Severity: The *IFNL4* rs368234815 DelG/DelG genotype was associated with risk for higher viral loads in COVID-19 patients (OR = 1.24, 95% CI 1.09–1.40).
Amoroso *et al*., 2021 [26]	Italian	219 cases /40,685 controls	*HLA-A*, *-B*, *-DRB1*	Disease susceptibility: The HLA-DRB1*08 allele was associated with risk for COVID-19 (OR = 1.9, 95% CI 1.2–3.1, P = 0.003) Severity: The HLA-DRB1*08 allele conferred risk for death (OR = 2.9, 95% CI 1.15–7.21, P = 0.023).
Avendaño-Félix *et al*., 2021 [27]	Mexican	193 cases	*IL-10*	Severity: The rs1800871 and rs1800872 polymorphisms were not associated with COVID-19 severity (P = 0.286 and P = 0.235, respectively) and related-outcomes (P = 0.499 and P = 0.531).
Benetti *et al*., 2020 [28]	Italian	131 cases /258 controls	*WES*	Disease susceptibility: *ACE2* allelic variability was higher in control group compared to the patient cohort, detected from a cumulative analysis of the identified variants (P <0.029).
Benetti *et al*., 2020 [29]	Italian	35 cases / 150 controls	*WES*	Disease susceptibility: Through the gene burden test, mutations in *PRKRA* and *LAPTM4B* genes were identified as being risk factors, while mutations in *OR4C5* and *NDU-FAF7* genes represented protective factors for COVID-19.
Bernas *et al*., 2021 [30]	German	4758 cases /10,5008 controls	*CCR5*	Disease susceptibility: The *CCR5* Δ*32* polymorphism was not associated with COVID-19 (OR = 0.96, 95% CI 0.89–1.03, P = 0.21). Severity: The *CCR5* Δ*32* polymorphism did not differ significantly between individuals with or without symptomatic infection (OR = 1.13, 95% CI 0.88–1.45, P = 0.32), severe respiratory tract infection (OR = 1.03, 95% CI 0.88–1.22, P = 0.68) or respiratory hospitalization (OR = 1.16, 95% CI 0.79–1.69, P = 0.45).
Cabrera-Marante *et al*., 2020 [31]	Latin-american, Spanish, Polish	22 cases	*PRF1*	Severity: Two of 22 patients showed *PRF1* A91V mutation in heterozygosis (allele frequency = 0.045). These 2 A91V-positive patients had higher fever associated with respiratory symptoms and died.
Cafiero *et al*., 2021 [32]	Italian	104 cases	*ACE1*, *ACE2*, *AGT*, *AGTR1*	Severity: The *ACE2* rs2074192 T, *ACE1* Del, and *AGT* rs699 C alleles were more frequent in symptomatic patients *vs*. asymptomatic (P = 0.001, P <0.001, and P = 0.033, respectively).
Calabrese *et al*., 2020 [33]	Italian	68 cases / 222 controls	*ACE1*	Severity: The frequency of *ACE1* Del/Del genotype was higher in COVID-19 patients with pulmonary embolism (PE) than patients without PE (72 *vs*. 46.5%; P = 0.048).
Cantalupo *et al*., 2021 [34]	Italy	202 cases /929 controls (rs35951367) 221 cases/1084 controls (rs3441865) 147 cases / 1095 controls (rs333)	*WES*	Disease susceptibility: The *CCR5* rs35951367 C allele was associated with risk for COVID-19 (OR = 1.307, 95% CI 1.01–1.70, P = 0.043). The *CCR5* rs34418657 G/T genotype was more frequent in patients with COVID-19 than controls (OR = 3.978, 95% CI 1.060–14.933, P = 0.027). No association was found between the *CCR5* Δ32 (rs333) polymorphism and COVID-19 (P = 0.99).
Coto *et al*., 2021 [35]	Spanish	318 cases / 350 controls	*ABO*	Disease susceptibility: The rs8176719 polymorphism was not associated with risk for COVID-19 or disease severity.
Cuesta-Llavona *et al*., 2021 [36]	Spanish	801 cases / 650 controls	*CCR5*	Disease susceptibility: Homozygosis for the *CCR5* Δ32 deletion (rs333) conferred protection against COVID-19 (OR = 0.66, 95% CI 0.49–0.88, P = 0.01).
Del Ser *et al*., 2021 [37]	Spanish	62 cases / 851 controls	*APOE*	Disease susceptibility: The *APOE* ε4 allele was associated with the presence of symptoms of COVID-19 (OR = 1.85, 95% CI 1.13–2.88, P = 0.010).
Dite *et al*., 2021 [38]	British	1582 cases^a^	*Array*	Severity: A score of 64 SNPs was associated with risk for COVID-19 severity (OR = 1.19, 95% CI 1.15–1.22, P <0.001). A model incorporating this score and clinical risk factors showed 111% better discrimination of disease severity than a model with just age and gender.
Ellinghaus *et al*., 2020 [39]	Italian, Spanish	835 cases / 1255 controls 775 cases/ 950 controls	*GSA*	Severity: The 3p21.31 cluster was identified as a susceptibility locus in patients with COVID-19 with respiratory failure (OR = 1.77, 95% CI 1.48–2.11; P = 1.15×10^−10^).
Gavriilaki *et al*., 2021 [40]	Greek	97 cases	*NGS*	Severity: Patients carrying the *THBD* rs1042580 C and *CFH* rs800292 G alleles did not require ICU hospitalization (*vs*. patients carrying the other alleles). Polymorphisms in *ADAMTS13*, *C3 and CFH* genes were associated with risk for ICU hospitalization (P = 0.022).
Gómez *et al*., 2020 [41]	Spanish	204 cases / 536 controls	*ACE1*, *ACE2*	Severity: The *ACE1* Del/Del genotype was associated with severe COVID-19 (P = 0.049). The *ACE2* rs2285666 polymorphism was not associated with disease severity.
Gómez *et al*., 2021 [42]	Spanish	311 cases / 440 controls	*IFITM3*	Disease susceptibility: The *IFITM3* rs12252 C allele was associated with risk for COVID-19 hospitalization after adjustment by age and gender (OR = 2.02, 95%CI 1.19–3.42, P = 0.01).
Grimaudo *et al*., 2021 [43]	Italian	383 cases	*MERTK*, *INFL4*, *PNPLA3*, *TLL1*	Severity: In patients younger than 65 years, the *PNPLA3* rs738409 G/G (OR = 4.69, 95% CI 1.01–22.04, P = 0.049) and *TLL1* rs17047200 T/T (OR = 9.1, 95% CI 1.45–57.3, P = 0.018) genotypes were associated with risk for disease severity.
Gunal *et al*., 2021 [44]	Turkish	90 cases	*ACE1*	Severity: The *ACE1* Ins/Ins genotype conferred protection against severe COVID-19 (OR = 0.323, 95% CI 0.112–0.929, P = 0.036).
Hamet *et al*., 2021 [45]	British	1644 cases / 15962 controls^a^	*Array*	Severity: The *ACE2* rs2074192 T allele was associated with more severe outcomes of COVID-19 in obese smoking males of 50 years or older (OR = 4.07, P = 0.036).
Hubacek *et al*., 2021 [46]	Czech	416 cases / 2404 controls^d^	*CCR5*	Severity: The frequency of CCR5 Δ32 allele was higher in COVID-19 asymptomatic patients (23.8%) than COVID-19-symptomatic patients (16.7%) (P = 0.03).
Hubacek *et al*., 2021 [47]	Czech	408 cases / 2559 controls^d^	*ACE1*	Disease susceptibility: The frequency of *ACE1* Ins/Ins genotype was higher in COVID-19 patients *vs*. controls (26.2% *vs*. 21.2%; OR = 1.55, 95% CI 1.17–2.05, P = 0.02).
Hubacek *et al*., 2021 [46]	Czech	408 cases / 2606 controls^d^	*APOE*	Disease susceptibility: The frequency of the APOE4 allele did not differ between the group of SARS-CoV-2-positive subjects and the control population (P = 0.11). Severity: The presence of least one *APOE4* allele was higher in symptomatic COVID-19 subjects than controls (OR = 1.43, 95% CI 1.05–1.95, P = 0.03). Genotype frequencies were almost identical in COVID-19-asymptomatic subjects and in the control group population (P = 0.86).
Karakas Çelik *et al*., 2021 [48]	Turkish	155 cases	*ACE1*, *ACE2*	Severity: *ACE1* Ins/Del and *ACE2* rs2106809 and rs2285666 polymorphisms were not associated with COVID‐19 severity.
Kerget *et al*., 2021 [49]	Turkish	70 cases	*IL-6*	Severity: The *IL-6* rs2074192 G/G genotype was associated with COVID-19 severity (P = 0.002).
Kolin *et al*., 2020 [50]	British	968 cases / 1734 controls^a^	*Array*	Disease susceptibility: Genome-wide association analysis did not show any significant loci in the meta-analysis (P >0.050).
Kuo *et al*., 2020 [51]	British	622 cases / 322326 controls^a^	*Array*	Disease susceptibility: The *ApoE* ε4ε4 genotype was associated with risk of COVID-19 positivity (OR = 2.24, 95% CI 1.72–2.93, P = 3.24 × 10^−9^) *vs*. e3e3 genotype. Severity: The presence of the *ApoE* ε4ε4 genotype conferred risk for mortality (OR = 4.29, 95% CI 2.38–7.72, P = 1.22 × 10^−6^) *vs*. e3e3 genotype.
Latini *et al*., 2020 [52]	Italian	131 cases / Controls^e^	*WES*	Disease susceptibility: *Furin* rs769208985 A and *TMPRSS2* rs114363287 A alleles were more frequent in COVID-19 than GnomAD controls (P = 0.005 and P = 0.016, respectively). *TMPRSS2* rs75603675 T and rs12329760 A alleles were less frequent in COVID-19 patients than GnomAD (P = 0.0446 and P = 0.023, respectively).
Lehrer *et al*., 2021 [53]	British	688 cases^a^	*S1R*	Severity: The *S1R* rs17775810 T/T genotype was associated with the lowest death rate (0%, P = 0.020).
Lehrer *et al*., 2021 [54]	British	712 cases / 9265 controls^a^	GWAS*-*Chr9	Disease susceptibility: No association was found between the rs657252 polymorphism in Chr9 and COVID-19.
Littera *et al*., 2020 [55]	Italian	182 cases / 619 controls	*HLA-A*, *-B*, *-C*, *-DRB1*	Disease susceptibility: The haplotype *HLA*-A*02:*05*, B*58:*01*, C*07:*01*, DRB1*03:*01* protected against SARS-CoV-2 infection. HLA-C*04:01 allele and the haplotype HLA-A*30:*02*, B*14:*02*, C*08:*02* (OR = 3.8, 95% CI 1.8–8.1, P = 0.025) were more frequent in patients than controls. Severity: HLA-DRB1*08:*01* allele was only present in hospitalized patients (OR >2.5, 95% CI 2.7–220.6, P = 0.024).
Lorente *et al*., 2020 [56]	Spanish	72 cases / 3,886 controls	*HLA-A*, *-B*, *-C*, *-DRB1*, *-DQB1*	Severity: The HLA-A*11, HLA-C*01 and HLA-DQB1*04 alleles were associated with higher mortality due to COVID-19 (OR = 7.69, 95% CI 1.06–55.65, P = 0.040; OR = 11.18, 95% CI 1.05–118.70, P = 0.040; and OR = 9.96, 95% CI 1.23–80.36, P = 0.030; respectively).
Malaquias *et al*., 2020 [57]	Brazilian	6 cases / 11 controls	*MBL2*	Disease susceptibility: The rs180040 A/A, rs1800451 G/G and rs5030737 C/C genotypes had a higher prevalence in the COVID-19 group.
Martínez-Sanz *et al*., 2021 [58]	Spanish	39 cases / 28 controls	*Array*	Disease susceptibility: The *ACE2* rs2106806 A (OR = 3.75, 95% CI 1.23–11.43, P = 0.015) and rs6629110 T (OR = 3.39, 95% CI 1.09–10.56, P = 0.028) alleles were associated with risk for COVID-19.
Medetalibeyouglu *et al*., 2021 [59]	Turkish	284 cases / 100 controls	*MBL2*	Disease susceptibility: The B/B genotype of the codon 54 A/B (Gly54Asp: rs1800450) variant in the *MBL2* gene was more frequent in COVID-19 cases *vs*. controls (10.9% *vs*. 1.0%; OR = 12.1, 95% CI 1.6–90.1, P = 0.001).
Möhlendick *et al*., 2021 [60]	Germany	297 cases / 253 controls	*ACE1*, *ACE2*	Disease susceptibility: The *ACE2* rs2285666 G/G genotype was associated with risk for COVID-19 (OR = 1.91, 95% CI 1.13–3.24, P = 0.02). No association was found between the *ACE1* rs1799752 polymorphism and COVID-19. Severity: The *ACE2* rs2285666 G/G genotype confer risk for serious course of COVID-19 compared to moderate course (OR = 3.04, 95% CI 1.47–6.27, P = 0.002) and is also associated with mortality (OR = 2.69, 95% CI 1.02–7.11, P = 0.05).
Monticelli *et al*., 2021 [61]	Italian	1177 cases^b^	*WES*	Severity: The *TMPRSS2* rs2298659 A and the rs12329760 T alleles were more frequent among mild cases of COVID-19 than severe cases (P = 0.004 and P = 0.029, respectively).
Naemi *et al*., 2021 [62]	Asian	95 cases	*HLA-A*, *-B*, *-C*, *-DRB1*, *-DQA1*, *-DQB1*	Severity: No association was found between these *HLA* genotypes and COVID-19 severity.
Novelli *et al*., 2020 [63]	Italian	131 cases / 1000 Controls^e^	*WES*	Disease susceptibility: No association was found between *ACE2* polymorphisms (rs140312271, rs2285666 and rs41303171) and COVID-19.
Novelli *et al*., 2020 [64]	Italian	99 cases / 1017 controls	*NGS*	Disease susceptibility: The frequencies of three *HLA* alleles were higher in cases *vs*. controls: HLA B*27:*07* (2.02% *vs*. 0.10%; P = 0.004), DRB1*15:*01* (10.10% *vs*. 4.62%, P = 0.048), and DQB1*06:*02* (7.58% *vs*. 3.64%, P = 0.016).
Pairo-Castineira *et al*., 2021 [65]		2244 cases^c^	*GWAS*	Severity: Polymorphisms in Chr 12q24.13 (rs10735079, P = 1.65 × 10^−8^, near to *OAS1*, *OAS2* and *OAS3* genes), Chr 19p13.2 (rs74956615, P = 2.3 × 10^−8^, near *TYK2*), Chr 19p13.3 (rs2109069, P = 3.98 × 10^−12^, in *DPP9*), and Chr 21q22.1 (rs2236757, P = 4.99 × 10^−8^, in *IFNAR2*) were associated with COVID-19 severity.
Petrazzuolo *et al*., 2020 [66]	French	140 cases	*FPR1*	Severity: No association was found between the *FPR1* rs5030880 and rs867228 polymorphisms and COVID-19 severity.
Posadas-Sánchez *et al*., 2021 [67]	Mexican	90 cases / 263 controls	*DPP4*	Disease susceptibility: The *DPP4* rs3788979 T/T genotype was associated with risk for COVID-19 (OR = 4.28, 95% CI 2.12–8.62, P = 4.7 × 10^−5^; recessive model).
Ravikanth *et al*., 2021 [68]	Indian	510 cases / 500 controls	*WES*	Severity: The *TMPRSS2* rs12329760 A allele was less frequent in patients with mild-to-moderate (P = 0.004) or severe disease (P = 0.010) *vs*. asymptomatic patients.
Russo *et al*., 2021 [69]	Italian	500 cases / 283 controls	*WES*	Severity: The *TNFRSF13* rs61756766 C allele was more frequent in severe cases *vs*. non-severe (OR = 11.5, 95% CI 1.3–100, P = 0.010) and asymptomatic patients (OR = 3.7, 95% CI 1.3–10.6, P = 0.020).
Saleh *et al*., 2021 [70]	Egyptian	900 cases / 184 controls	*TNFA*	Disease susceptibility: The A/A genotype of the *TNF* G308A polymorphism was associated with risk for COVID-19 (OR = 3.06, 95% CI 1.26–7.44, P = 0.019).
Salem Hareedy *et al*., 2021 [71]	Egyptian	46 cases / 14 controls	CYP2D6*4, CYP2D6*2XN, CYP3A4*1B, CYP3A5*3	Disease susceptibility: Carriers of the CYP2D*2XN C/C genotype had the lower risk for a positive anti-COVID-19 IgG or IgM. The CYP3A4*1B A/A genotype conferred protection against positive anti-COVID-19 IgM (*vs*. G/G genotype).
Schönfelder *et al*., 2021 [72]	Germany	239 cases / 253 controls	*IFITM3*	Disease susceptibility: The *IFITIM3* rs12252 and rs34481144 polymorphisms were not associated with COVID-19 development (OR = 1.37, 95% CI 0.73–2.58, P = 0.340; OR = 0.96, 95% CI 0.65–1.41, P = 0.840; respectively). Severity: The *IFITIM3* rs12252 and rs34481144 polymorphisms did not confer risk to COVID-19 severity (OR = 0.89, 95% CI 0.35–2.25, P = 1.00; OR = 1.77, 95% CI 0.94–3.32, P = 0.100; respectively).
Schönfelder *et al*., 2021 [73]	Germany	239 cases / 253 controls	*TMPRSS2*	Disease susceptibility: The *TMPRSS2* rs383510 C/C genotype was associated with risk for COVID-19 infection (OR = 1.73, 95% CI 1.15–2.59, P = 0.010). The rs2070788 and rs12329760 polymorphisms were not associated with COVID-19.
Scutt *et al*., 2021 [74]	British	705 cases / 471506 controls^a^	*Array*	Disease susceptibility: The *INK4A/ARF* rs10757278 G/A genotype was associated with lower risk of hospital admission for COVID-19 in non-Caucasian patients (A/A + G/G *vs*. A/G; OR = 0.56, 95% CI 0.37–0.85, P = 0.006).
Shikov *et al*., 2020 [75]	Russian	37 cases /21 controls	*ACE2*, *ACE1*	Disease susceptibility: No association was found between *ACE2* and *ACE1* polymorphisms and COVID-19.
Shkunikov *et al*., 2021 [76]	Russian	111 cases / 428 controls	*NGS*	Disease susceptibility: The HLA-A*01:*01* allele was associated with risk for COVID-19, while the HLA-A*02:*01* and HLA-A*03:*01* alleles conferred protection.
Torre-Fuentes *et al*., 2021 [77]	Spanish	4 cases / 71 controls	*WES*	Disease susceptibility: No association was found between *ACE2*, *TMPRSS2* and *FURIN* polymorphisms and COVID-19.
Valenti *et al*., 2021 [78]	Spanish	72 cases	*Chr3*	Severity: The rs11385942 G/A genotype was associated with COVID-19 severity.
Verma *et al*., 2021 [79]	Indian	269 cases	*ACE1*	Severity: The *ACE1* Del/Del genotype was associated with risk for severe COVID-19 (OR = 3.69, 95% CI 1.612–8.431, P = 0.002).
Vietzen *et al*., 2021 [80]		361 cases / 260 controls	*HLA-E*, *KLRC2*	Disease susceptibility: The *KLRC2* Del allele conferred risk for hospitalization (OR = 2.6, P = 0.0006) and hospitalization in ICU (OR = 7.1, P <0.0001) *vs*. non-hospitalized patients and controls. Severity: The HLA-E*0101 allele was also associated with risk for hospitalization (OR = 2.1, P = 0.010) and hospitalization in ICU (OR = 2.7, P = 0.010).
Wang *et al*., 2020 [81]	Chinese	332 cases	GWAS* / *HLA-A*, *-B*, *-C*, *-DRB1*, *-DQB1*, *-DPB1*, *-DQA1*	Severity: The *TMEM189–UBE2V1* rs6020298 A allele was more frequent in patients with severe COVID-19 than non-severe patients (0.59 *vs*. 0.45) and conferred risk for mild + severe disease (OR = 1.2, P = 4.1 x 10^−6^). The *TMPRSS2* rs12329760 minor allele was less frequent among patients with severe COVID-19 *vs*. mild symptomatic patients. HLA-A* 11:*01*, B*51:*01*, and C*14:*02* alleles were associated with risk for severe COVID-19.
Wang *et al*., 2020 [82]	Chinese	82 cases / 3548 controls	*NGS*	Disease susceptibility: HLA-B*15:*27* and HLA-C*07:29 were associated with risk for COVID-19 disease (OR = 3.59; 95% CI 1.72–7.50, P = 0.030; and OR = 130.20, 95% CI 5.28–3211, P = 0.025, respectively).
Wulandari *et al*., 2021 [83]	Indonesian	95 cases	*TMPRSS2*	Severity: No association was found between the rs12329760 polymorphism and COVID-19 severity.
Zhang *et al*., 2020 [84]	China	80 cases	*IFITM3*	Severity: The *IFITM3* rs12252 C/C genotype was associated with disease severity in an age-dependent manner (OR = 6.37, P <0.001).
Zhou *et al*., 2020 [85]	British	1091 cases / 2793 controls^a^	*TMPRSS2*, *ACE2*	Disease susceptibility: After analyzing 17 and 31 tag SNPs of *ACE2* and *TMPRSS2* genes, respectively, the rs7282236 SNP in *TMPRSS2* gene was the only one associated with risk of COVID-19 disease (OR = 1.33, 95% CI 1.14–1.54, P = 2.31 × 10^−4^).

Chr: chromosome; GSA: Global Screening Array; GWAS: Genome-wide Association Study; ICU: intensive care unit, NGS: next-generation sequencing; WES: Whole exome sequencing

^a^data from UK biobank

^b^data from GEN-COVID Multicenter Study

^c^data from GenOMICC database

^d^controls data from post-MONICA study

^e^controls data from GnomAD database.

### Qualitative synthesis of studies that analyzed associations of SNPs and COVID-19

**Table 1** shows the compiled main data of the 64 eligible studies included in this systematic review. More than 200 polymorphisms and 50 genes/loci were studied regarding their associations with COVID-19 susceptibility or severity of this disease. Most of the studies compared polymorphism frequencies in patients who tested positive for COVID-19 compared to negative controls. Twenty-three studies evaluated polymorphisms in COVID-19 patients categorized according to different degrees of disease severity. **S1 Table** shows the quality of all studies included in this systematic review, which was evaluated using the CBS as described in the Methods Section. Considering a score system that ranges from 0 to 10 points according to the adherence to pre-defined criteria, none of the studies reached 9 points. However, the majority of the studies (70.1%) were classified as presenting good quality since they were awarded 6 to 8 points. The remaining articles were awarded with less than 6 points.

More information regarding the COVID-19 diagnostic criteria, definition of severity degrees, age, ethnicity, gender, and genotyping techniques are described in **S2 Table.** The most studied candidate genes/loci were: *HLA*, *ABO*, *ACE1*, *ACE2*, *APOE*, *CCR5*, *TMPRSS2*, and *IFITM3*. In total, 8 polymorphisms in 7 candidate genes and 74 alleles of the *HLA* loci (*A*, *B*, *C*, *DRB1*, *DQA1*, and *DQB1*) were analyzed in ≥3 studies and subsequently included in the meta-analyses.

### Meta-analyses of *ACE2*, *ACE1*, and *TMPRSS2* polymorphisms

Two polymorphisms in the *ACE2* gene were included in meta-analyses (**Table 2**). The pooled data of 3 studies for the rs41303171 (T/C) polymorphism [28, 63, 77] and 3 studies for the rs2285666 (C/T) polymorphism [41, 58, 63] indicated no association between them and the risk for COVID-19.

**Table 2:** Meta-analyses of the association between polymorphisms in candidate genes and COVID-19 development and severity.

Polymorphism	Localization/Position	Inheritance model	Studies	I^2^	Model	OR (95% CI)
**COVID-19 infection *vs*. Control**						
*ACE2* rs2285666	chrX:15592225 / Intron	Dominant	3	64.1%	Random	0.95 (0.57–1.56)
*ACE2* rs41303171	chrX:15564175 / Exon	Allele	3	66.3%	Random	1.52 (0.24–9.61)
		Dominant	3	67.8%	Random	1.36 (0.20–9.20)
*ACE1 Ins/Del*	chr17:63488530–63488543 / Intron	Allele	4	61.7%	Random	1.00 (0.82–1.22)
		Dominant	4	64.1%	Random	0.95 (0.70–1.28)
		Recessive	4	64.2%	Random	0.93 (0.64–1.37)
		Additive	4	72.3%	Random	0.89 (0.55–1.46)
*TMPRSS2* rs12329760	chr21:41480570 / Exon	Allele	3	12.6%	Fixed	1.08 (0.92–1.27)
		Dominant	3	0%	Fixed	1.18 (0.96–1.45)
*CCR5* rs333	chr3:46373453–46373487 / Exon	Allele	3	44.6%	Fixed	0.80 (0.68–0.96)*
		Dominant	3	40.3%	Fixed	0.82 (0.68–0.98)*
*ApoE* ε4	chr19:44908684 and chr19:44908822† / Exon	Allele	3	41.8%	Fixed	1.32 (1.20–1.45)*
		Dominant	3	58.2%	Random	1.38 (1.09–1.75)*
		Recessive	3	28.2%	Fixed	1.94 (1.50–2.50)*
		Additive	3	27.1%	Fixed	2.05 (1.58–2.65)*
*ABO* rs8176719	chr9:133257521–133257522 / Exon	Allele	3	80.7%	Random	1.22 (0.99–1.49)
**COVID-19 mild/moderate *vs*. severe**						
*ACE1Ins/Del*	chr17:63488530–63488543 / Intron	Allele	5	45.4%	Fixed	0.67 (0.56–0.82)*
		Dominant	5	41.4%	Fixed	0.62 (0.47–0.83)*
		Recessive	5	0%	Fixed	0.69 (0.50–0.95)*
		Additive	5	0%	Fixed	0.49 (0.33–0.72)*
*TMPRSS2* rs12329760	chr21:41480570 / Exon	Allele	5	0%	Fixed	0.77 (0.66–0.91)*
		Dominant	5	0%	Fixed	0.74 (0.61–0.90)*
		Recessive	5	0%	Fixed	0.71 (0.44–1.15)
		Additive	5	0%	Fixed	0.65 (0.40–1.06)
*CCR5* rs333	chr3:46373453–46373487 / Exon	Allele	3	67.2%	Random	0.83 (0.59–1.16)
		Dominant	3	67.4%	Random	0.83 (0.58–1.18)
*IFITM3* rs12252	chr11:320772 / Exon	Allele	4	65.6%	Random	1.04 (0.62–1.75)
		Dominant	4	65.8%	Random	0.97 (0.53–1.77)
		Recessive	4	22.5%	Fixed	1.04 (0.44–2.46)
		Additive	4	34.3%	Fixed	0.78 (0.31–1.91)
*ApoE* ε4	chr19:44908684 and chr19:44908822† / Exon	Allele	3	0%	Fixed	1.36 (1.07–1.73)*
		Dominant	3	0%	Fixed	1.30 (0.97–1.72)
*ABO* rs8176719	chr9:133257521–133257522 / Exon	Allele	4	0%	Fixed	0.94 (0.85–1.05)

OR: odds ratio; CI: confidence interval.

* Indicates a significant association at P <0.05.

† Location of the two polymorphisms (rs429358 and rs7412) that generated the *ApoE* ε4 haplotype.

**Fig 2:**
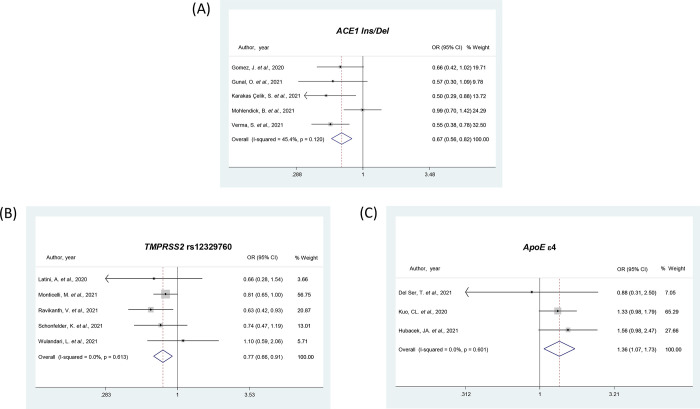
Forest plots showing individual and pooled ORs (95% CIs) for the associations between the *ACE1 Ins/Del* (**A**), *TMPRSS2* rs12329760 (**B**), and *ApoE* ε4 (**C**) polymorphisms and COVID-19 severity, under the allele contrast model.

The rs1799752 (Ins/Del) polymorphism in the *ACE1* gene was analyzed in 4 studies [33, 41, 47, 60] and the meta-analysis indicated no association between the Ins allele and the risk for COVID-19 (**Table 2**). Regarding COVID-19 severity, 8 studies [32, 33, 44, 47, 48, 58, 60, 79] were included. However, we analyzed the pooled data from 5 studies [41, 44, 48, 60, 79] that included severe COVID-19 patients compared to other degrees of severity (moderate, mild and/or asymptomatic). The meta-analysis of these studies showed an association between the *ACE1* rs1799752 Ins allele and protection against the most severe form of COVID-19, in all inheritance models (OR = 0.67, 95% CI 0.56–0.82, **Table 2** and **Fig 2A** for the allele model). Hubacek *et al*. [47] and Cafiero *et al*. [32] studies only compared asymptomatic *vs*. symptomatic patients, while the study be Calabrese *et al*. [33] compared groups according to the presence of thromboembolism in patients with severe COVID-19. Of note, when we included all the 8 studies in the meta-analysis, the Ins allele remained associated with protection against severe COVID-19 (OR = 0.60, 95% CI 0.39–0.94, for the allele model).

The *TMPRSS2* rs12329760 (C/T) polymorphism was analyzed in 3 studies regarding COVID-19 infection [52, 68, 73, 77] and 5 studies investigating disease severity [61, 68, 73, 83] (**Table 2**). Although the rs12329760 polymorphism was not associated with the risk of COVID-19, this meta-analysis showed that the T allele of this polymorphism confers protection for the most severe form of COVID-19 when considering both allele (OR = 0.77, 95% CI 0.66–0.91; **Fig 2B**) and dominant model (OR = 0.74, 95% CI 0.61–0.90) models (**Table 2**).

### Meta-analyses of *HLA* alleles

**Fig 3:**
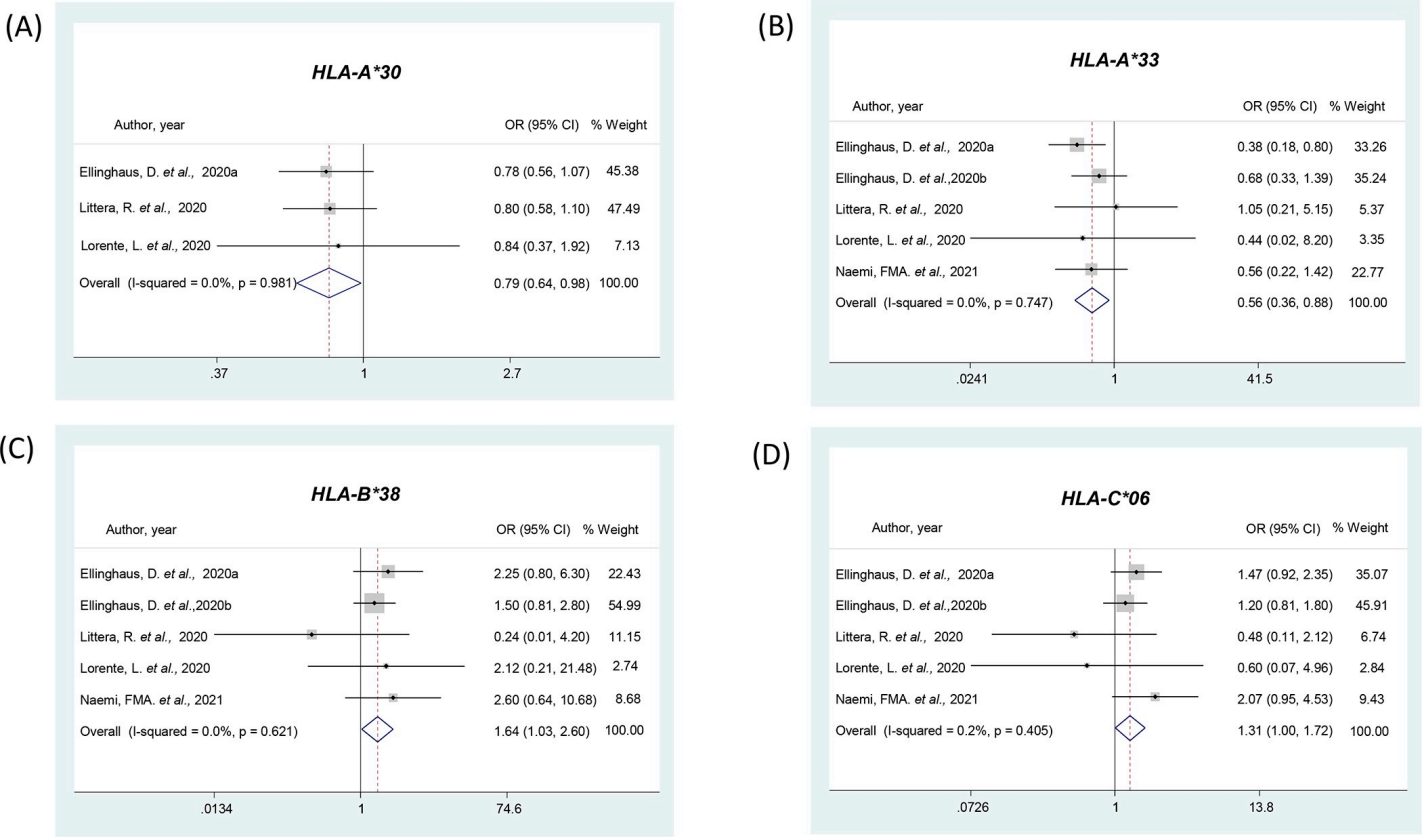
Forest plots showing individual and pooled ORs (95% CIs) for the associations between *HLA* alleles and COVID-19 presence or severity. **(A)** Forest plot for HLA-A*30 and COVID-19 presence. **(B)** Forest plot for HLA-A*33 and COVID-19 severity. **(C)** Forest plot for HLA-B*38 and COVID-19 severity. **(D)** Forest plot for HLA-B*06 and COVID-19 severity. ^a^ Data from an Italian population; ^b^ Data from a Spanish population.

The *A*, *B*, *C*, *DRB1*, *DQB1*, and *DQA1* alleles of the *HLA* were analyzed according to the risk of COVID-19 (**S3 Table**) or the severity of the disease (**S4 Table**). The HLA-A*30 allele was analyzed in 3 studies [39, 55, 56], and the pooled analysis showed this allele confers protection against COVID-19 (OR = 0.79, 95% CI 0.64–0.98; **S3 Table** and **Fig 3A**).

Regarding COVID-19 severity, the pooled data of 4 articles (5 studies) [39, 55, 56, 62] showed the association between the HLA-A*33 allele and protection for the most severe form of disease (OR = 0.56, 95% CI 0.36–0.88; **S4 Table** and **Fig 3B**). In contrast, the HLA-B*38 and HLA-C*06 alleles, both analyzed in the same 4 articles (5 studies) [39, 55, 56, 62], were associated with risk for the most severe form of COVID-19 (OR = 1.64, 95% CI 1.03–2.60 and OR = 1.31, 95% CI 1.00–1.72, respectively; **S4 Table** and **Fig 3C and 3D**). Our meta-analyses demonstrated that the other 70 alleles of the *A*, *B*, *C*, *DRB1*, *DQB1*, and *DQA1* loci were not associated with COVID-19 development or severity (**S3** and **S4 Tables**).

### Meta-analyses of *CCR5* and *IFITM3* polymorphisms

**Fig 4:**
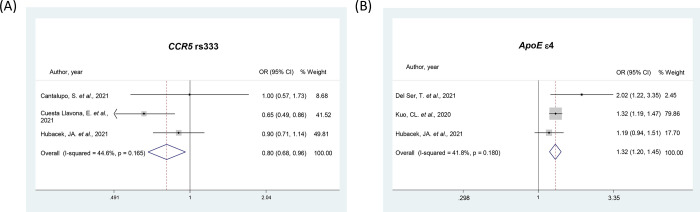
Forest plots showing individual and pooled ORs (95% CIs) for the associations between the *CCR5* rs333 (**A**) and *ApoE* ε4 (**B**) polymorphisms and COVID-19 presence, both under the allele contrast model.

Three studies were included in the meta-analyses of *CCR5* rs333 (Ins/Del) polymorphism regarding the risk of COVID-19 and its severity [30, 36, 46] (**Table 2**). The Del allele was associated with protection for COVID-19 infection considering both allele (OR = 0.80, 95% CI 0.68–0.96; **Fig 4A**) and dominant (OR = 0.82, 95% CI 0.68–0.98) models; however, this polymorphism was not associated with the severity of the disease (**Table 2**).

For the *IFITM3* rs12252 (T/C) polymorphism, the pooled analyses of 4 studies [24, 42, 72, 84] indicated no association of this polymorphism and different degrees of COVID-19 severity, for all tested genetic models (**Table 2**).

### Meta-analyses of *ApoE* and *ABO* polymorphisms

The *ApoE* ε4 genotype was analyzed in 3 studies [37, 46, 51] regarding both COVID-19 infection and severity (**Table 2)**. Meta-analyses showed the ε4 allele was associated with risk for COVID-19 presence in all genetic models (OR = 1.32, 95% CI 1.20–1.45, **Fig 4B** for the allele model). The ε4 allele was also associated with risk for the most severe form of COVID-19 when considering the allele model (OR = 1.36, 95% CI 1.07–1.73, **Fig 2C**).

The rs8176719 (-/C) polymorphism in the *ABO* gene was evaluated in 3 studies (2 articles) [38, 39] about COVID-19 development and 4 studies (3 articles) regarding disease severity [35, 38, 39] (**Table 2**). The pooled analyses indicated the Ins C allele is not associated with COVID-19 presence or severity in the allele model.

## Discussion

Elucidating the genetic determinants of SARS-CoV-2 infection is essential for understanding the pathophysiology of COVID-19 and the inter-individual variability in its severity; thus, contributing to the development of updated vaccines and new antivirals. Hence, in this systematic review, we summarized the results of 64 eligible articles that analyzed the association between genetic polymorphisms and risk for infection or severity of COVID-19. Moreover, data regarding polymorphisms in 8 genes (*HLA*, *ABO*, *ACE1*, *ACE2*, *APOE*, *CCR5*, *TMPRSS2*, and *IFITM3)* were meta-analyzed in relation to the risk of infection and severity of COVID-19. Pooled results demonstrated that polymorphisms in the *ApoE*, *ACE1*, *TMPRSS2*, *CCR5*, and *HLA* genes appear to be involved in the susceptibility to and/or severity of COVID-19.

Angiotensin-converting enzyme 2 (*ACE2*) and type II transmembrane serine protease (*TMPRSS2*) are candidate genes for susceptibility for SARS-CoV-2 infection since SARS-CoV-2 uses the ACE2 receptor for cell entry, while the serine protease TMPRSS2 is required for priming of the viral spike (S) protein [86, 87]. ACE2 and ACE1, together with renin and angiotensin, constitute the renin angiotensin aldosterone system (RAAS), which is a complex system involved in multiple biological process that regulated blood pressure homeostasis and extracellular volume, and inflammation, which is closely related to COVID-19 morbidity and mortality, as it affects bradykinin production [88, 89]. Following the viral entry, ACE2 is down-regulated, causing an ACE1/ACE2 imbalance and contributing to RAAS overactivation and pulmonary shutdown. The consequent increased ACE1 activity and reduced ACE2 expression increase the risk of pulmonary diseases by increasing the lung vascular permeability; thus, leading to lung damage [90–92]. Accordingly, studies have reported the association between polymorphisms in *ACE1*, *ACE2*, and *TMPRSS2* genes and SARS-CoV-2 infection [28, 32, 33, 41, 44, 48, 52, 58, 60, 61, 63, 68, 73, 77, 83]; however, the results are still contradictory. In the present meta-analysis, two *ACE2* polymorphisms (rs2285666 and rs41303171) were analyzed, but no association with COVID-19 was found. Nevertheless, we demonstrated an association between the T allele of the *TMPRSS2* rs12329760 polymorphism and protection against the most severe form of COVID-19.

Regarding the *ACE1* gene, the insertion/deletion (Ins/Del) of 287-bp in the *Alu*-sequence of intron 16, represented by four individual SNPs (rs4646994, rs1799752, rs4340 and rs13447447), modulates *ACE1* expression [93–95]. This Ins/Del variant results in alternative splicing, leading to protein shortening and loss of the catalytically active domain in *ACE1* Ins allele carriers [92]. Moreover, the *ACE1* Ins/Del variant explains about 60% of variability in ACE1 levels in the general population since ACE1 levels in Ins/Ins carriers are approximately half of that of Del/Del carriers [39, 93, 96]. In the context of SARS-CoV-2 infection, studies have reported variations in COVID-19 recovery and prevalence rates are associated to *ACE1* Ins/Del frequency and geographical variations of this variant [97, 98]. Here, we showed an association between the *ACE1* Ins allele and protection against severe COVID-19.

Major histocompatibility complex genes (*MHC*, known as Human Leukocyte Antigens, *HLA*) play a critical role in immune response [99]. The HLA system is a remarkably polymorphic region and genetic variants of *HLA* have been reported to affect the clinical course of patients infected with different viruses [100], including SARS-CoV-1 [101]. A specific set of HLA will present the peptides of the degraded virus to receptors on T cells, thus eliciting an immune response for virus eradication [102]. The set of *HLA* alleles inherited by an individual will determine the immune responses to viruses according to the selected peptides that can bind to the peptide‐binding groove [102]. Studies in different populations have shown associations between some *HLA class I* (*A*, *B*, and *C*) and *class II* (*DRB1*, *DQA1*, and *DQB1*) alleles and COVID-19 susceptibility and/or severity [82, 103]. Our meta-analyses did not confirm the results of previous individual studies; however, we identified new *HLA* alleles associated with COVID-19: the HLA-A*30 and HLA-A*33 were associated with protection against COVID-19 infection and the most severe form of this disease, respectively. Besides, the HLA-B*38 and HLA-C*06 alleles were associated with risk for severe COVID-19.

The interferon-induced transmembrane 3 (IFITM3) is an IFN-stimulated gene (ISG) essentially expressed on endosomes and lysosomes [104]. IFITM3 is part of an ISG family (IFITM) responsible for inhibiting the fusion between viral and cellular membranes of many viruses, such as influenza A H1N1 virus, dengue virus, and SARS-CoV [104]. On the other hand, it was recently shown that IFITM proteins are cofactors for efficient SARS-CoV-2 infection in human cells [105], reaffirming a key role of this gene in the susceptibility to COVID-19. Nevertheless, here, the *IFITM3* rs12252 polymorphism was not associated with COVID-19 severity. Of note, we did not analyze this polymorphism regarding COVID-19 infection susceptibility due to lack of studies. Although this SNP in *IFITM3* gene was not associated with COVID-19, it is noteworthy that type I IFN (IFN-I)-stimulated immunity has been shown to influence COVID-19 severity. Inborn errors of IFN-I pathway and pre-existing auto-antibodies neutralizing IFN-I appear to be strong determinants of critical COVID-19 pneumonia in about 15–20% of patients [106]. Asano *et al*., [107] reported that deleterious X-linked *TLR7* mutations were observed in 16 male subjects from a cohort of 1202 patients with unexplained critical COVID-19 pneumonia. The patients’ blood plasmacytoid dendritic cells (pDCs) produced low levels of IFN-I in response to SARS-CoV-2. Human TLR7 and pDCs are essential for protective IFN-I immunity against SARS-CoV-2 in the respiratory tract. Moreover, Zhang *et al*., [108] showed that inborn errors of TLR3- and IRF-7 dependent IFN-I immunity can cause life-threatening COVID-19 pneumonia in patients with no prior severe infection.

Chemokines act attempting to maintain the immune homeostasis and to defend the body against harmful stimuli, such as SARS-CoV-2 infection [109]. *CCR5* encodes a chemokine receptor expressed in macrophages and T cells, and its upregulation has been confirmed in COVID-19 patients [110]. Furthermore, an anti-CCR5 treatment has been shown to relieve the symptoms and the cytokine storm in COVID-19 patients who are critically ill [109]. The *CCR5* gene is located at 3p21.31, a gene cluster region associated with severe COVID-19 courses [39]. The most studied *CCR5* polymorphism regarding COVID-19 susceptibility is the Δ32 Ins/Del (rs333) [30, 34, 36, 46]. The *CCR5* rs333 Del allele results in loss of function of the protein; being a major determinant of the resistance to HIV infection since the CCR5 protein serves as one of the gateways for the HIV virus [111]. Accordingly, our meta-analysis showed the *CCR5* rs333 Del allele was associated with protection against COVID-19 infection [34, 36, 46].

A Genome-Wide Association Study (GWAS) carried out by the Severe COVID-19 GWAS Group [39] reported that one of the 2 strongest signals associated with severe COVID-19 was located within the ABO blood-group system. The involvement of ABO blood groups in COVID-19 susceptibility has been reported in both genetic and non-genetic studies. The blood group O was previously associated with a lower risk of acquiring COVID-19 when compared to subjects with non-O blood groups, whereas the blood A group was associated with a higher risk for this disease than non-A blood groups [39]. One of the assumptions is that the A-antigen causes P-selectin and intercellular cell adhesion molecule 1 binding to endothelial cells, increasing the probability of cardiovascular disease. Another explanation is that individuals with blood group O have decreased levels of von Willebrand factor, lowering the thrombotic disease risk [reviewed in [103]]. The rs8176719 polymorphism is the main determinant of the O blood group and has been investigated as a potential marker of COVID-19 susceptibility. However, some studies did not confirm these findings [35, 38]. In our meta-analysis, we demonstrated that the *ABO* rs8176719 - /C SNP was not associated with COVID-19 infection neither with different stages of severity.

The *ApoE* ε4 genotype was investigated in the UK Biobank Cohort, being associated with COVID-19 severity and mortality [51]. This finding was replicated in other studies [37, 46]. Apolipoprotein E (ApoE) is broadly expressed in human tissues and has an essential role in lipid transport, which has a key role in many functions, including immunity [112]. The most studied polymorphisms in *ApoE* are the rs429358 (ApoE4, C/T) and rs7412 (ApoE2, C/T), both located at exon 4. Three haplotypes are generated from these two polymorphisms (ε2, ε3 and ε4), codifying 3 protein isoforms (E2, E3 and E4). Moreover, these haplotypes can combine in 6 different variants: ε2/ε2, ε2/ε3, ε2/ε4, ε3/ε3, ε3/ε4, and ε4/ε4 [112]. Among them, the ancestral *ApoE* ε4/ε4, generally considered deleterious, is a significant risk factor for Alzheimer’s disease and other human pathologies, including type 2 diabetes and cardiovascular disease, which are known risk factors for worst outcomes of COVID-19 [112–114]. In the present meta-analysis, the pooled data of three studies confirmed the association of the ε4 allele with both risk to COVID-19 presence and severe outcomes of the disease. It has been hypothesized that elevated cholesterol and oxidized lipoprotein levels, linked to the effects of ApoE ε4/ε4 variant, is associated with increased pneumocyte susceptibility to infection and to exaggerated lung inflammation [112]. Moreover, the frequency of the ε4 allele is higher in African-Americans who had increased mortality due to COVID-19 compared to Caucasian populations [115].

The results of the present meta-analysis should be interpreted within the context of a few limitations. Inter-studies heterogeneity is common in meta-analyses of genetic association studies and it should be cautiously interpreted. Some included studies did not test the control groups for COVID-19 or included controls derived from previous databank or ecological studies without COVID-19 information. Moreover, the COVID-19 severity criteria varied among the studies. Particular studies had included asymptomatic patients while others only included patients with at least a given symptom. Due to the presence of more than 2 groups of COVID-19 severity stages (mild, moderate and severe), we have categorized the patients regarding COVID-19 severity in different ways; however, it was more rational to show the data categorizing the most severe group against the others groups (asymptomatic and/or mild plus moderate). It was not possible to evaluate the association with mortality, as only few studies presented data comparing COVID-19 survivors and non-survivors. Furthermore, the impact of gender and age, which may influence the COVID-19 predisposition, could not be assessed due to the small number of studies for each SNP. Genetic background among different populations may significantly influence COVID-19 susceptibility, and the studies included in the present meta-analysis comprised different ethnicities. However, due to the small number of studies for each ethnicity, we were not able to analyze the impact of genetic background on the results. Finally, we cannot be sure that small negative studies were overlooked since we could not perform the publication bias analysis due to the small amount of studies for each SNP.

The infection with SARS-CoV-2 and its clinical course are dependent on the complex relationship between the virus and the host immune system. In this meta-analysis, we identified, for the first time, that four alleles of the *HLA class I* loci (A*30, A*33, B*38 and C*06) are associated with COVID-19. Moreover, we confirmed the association between COVID-19 susceptibility and polymorphisms in the *ApoE*, *ACE1*, *TMPRSS2*, and *CCR5* genes. These findings will guide further epidemiological studies on host genetics as well as the development of innovative treatments. Considering that specific genetic polymorphisms might lead to severe COVID-19 outcomes, it is of extreme importance to use individual genetic data to employ personalized therapeutics and improve the COVID-19 prognostic.

## Supporting information

S1 Table Clark-Baudouin quality assessment scale for the studies included in the systematic-review. (DOCX)

S2 Table Characteristics of studies included in this systematic review and meta-analysis. (XLSX)

S3 Table Meta-analyses of the association between polymorphisms in HLA and COVID-19. (DOCX)

S4 Table Meta-analyses of the association between polymorphisms in *HLA* and COVID-19 severity. (DOCX)

## References

[1] Guo G, Ye L, Pan K, Chen Y, Xing D, Yan K, et al. New Insights of Emerging SARS-CoV-2: Epidemiology, Etiology, Clinical Features, Clinical Treatment, and Prevention. Front Cell Dev Biol. 2020;8:410. doi: 10.3389/fcell.2020.00410 32574318 PMC7256189

[2] Alshoabi SA, Alhazmi FH, Abdulaal OM, Gameraddin MB, Algaberi AK, Hamid AM, et al. Frequent clinical and radiological manifestations of the Novel SARS-CoV-2: A review article. J Family Med Prim Care. 2021;10(1):122–6. doi: 10.4103/jfmpc.jfmpc_1985_20 34017713 PMC8132777

[3] Atzrodt CL, Maknojia I, McCarthy RDP, Oldfield TM, Po J, Ta KTL, et al. A Guide to COVID-19: a global pandemic caused by the novel coronavirus SARS-CoV-2. FEBS J. 2020;287(17):3633–50. doi: 10.1111/febs.15375 32446285 PMC7283703

[4] Nakeshbandi M, Maini R, Daniel P, Rosengarten S, Parmar P, Wilson C, et al. The impact of obesity on COVID-19 complications: a retrospective cohort study. Int J Obes (Lond). 2020;44(9):1832–7. doi: 10.1038/s41366-020-0648-x 32712623 PMC7382318

[5] Zhou F, Yu T, Du R, Fan G, Liu Y, Liu Z, et al. Clinical course and risk factors for mortality of adult inpatients with COVID-19 in Wuhan, China: a retrospective cohort study. Lancet. 2020;395(10229):1054–62. doi: 10.1016/S0140-6736(20)30566-3 32171076 PMC7270627

[6] Anastassopoulou C, Gkizarioti Z, Patrinos GP, Tsakris A. Human genetic factors associated with susceptibility to SARS-CoV-2 infection and COVID-19 disease severity. Hum Genomics. 2020;14(1):40. doi: 10.1186/s40246-020-00290-4 33092637 PMC7578581

[7] Elhabyan A, Elyaacoub S, Sanad E, Abukhadra A, Elhabyan A, Dinu V. The role of host genetics in susceptibility to severe viral infections in humans and insights into host genetics of severe COVID-19: A systematic review. Virus Res. 2020;289:198163. doi: 10.1016/j.virusres.2020.198163 32918943 PMC7480444

[8] Grolmusz VK, Bozsik A, Papp J, Patocs A. Germline Genetic Variants of Viral Entry and Innate Immunity May Influence Susceptibility to SARS-CoV-2 Infection: Toward a Polygenic Risk Score for Risk Stratification. Front Immunol. 2021;12:653489. doi: 10.3389/fimmu.2021.653489 33763088 PMC7982482

[9] Debnath M, Banerjee M, Berk M. Genetic gateways to COVID-19 infection: Implications for risk, severity, and outcomes. FASEB J. 2020;34(7):8787–95. doi: 10.1096/fj.202001115R 32525600 PMC7300732

[10] Ramos-Lopez O, Daimiel L, Ramirez de Molina A, Martinez-Urbistondo D, Vargas JA, Martinez JA. Exploring Host Genetic Polymorphisms Involved in SARS-CoV Infection Outcomes: Implications for Personalized Medicine in COVID-19. Int J Genomics. 2020;2020:6901217. doi: 10.1155/2020/6901217 33110916 PMC7582067

[11] Ozturk R, Tasova Y, Ayaz A. COVID-19: pathogenesis, genetic polymorphism, clinical features and laboratory findings. Turk J Med Sci. 2020;50(SI-1):638–57. doi: 10.3906/sag-2005-287 32512673

[12] SeyedAlinaghi S, Mehrtak M, MohsseniPour M, Mirzapour P, Barzegary A, Habibi P, et al. Genetic susceptibility of COVID-19: a systematic review of current evidence. Eur J Med Res. 2021;26(1):46. doi: 10.1186/s40001-021-00516-8 34016183 PMC8135169

[13] Moher D, Liberati A, Tetzlaff J, Altman DG, Group P. Preferred reporting items for systematic reviews and meta-analyses: the PRISMA statement. BMJ. 2009;339:b2535. doi: 10.1136/bmj.b2535 19622551 PMC2714657

[14] Stroup DF, Berlin JA, Morton SC, Olkin I, Williamson GD, Rennie D, et al. Meta-analysis of observational studies in epidemiology: a proposal for reporting. Meta-analysis Of Observational Studies in Epidemiology (MOOSE) group. JAMA. 2000;283(15):2008–12. doi: 10.1001/jama.283.15.2008 10789670

[15] Sagoo GS, Little J, Higgins JP. Systematic reviews of genetic association studies. Human Genome Epidemiology Network. PLoS Med. 2009;6(3):e28. doi: 10.1371/journal.pmed.1000028 19260758 PMC2650724

[16] de Souza BM, Brondani LA, Boucas AP, Sortica DA, Kramer CK, Canani LH, et al. Associations between UCP1 -3826A/G, UCP2 -866G/A, Ala55Val and Ins/Del, and UCP3 -55C/T polymorphisms and susceptibility to type 2 diabetes mellitus: case-control study and meta-analysis. PLoS One. 2013;8(1):e54259. doi: 10.1371/journal.pone.0054259 23365654 PMC3554780

[17] Brondani LA, Assmann TS, de Souza BM, Boucas AP, Canani LH, Crispim D. Meta-analysis reveals the association of common variants in the uncoupling protein (UCP) 1–3 genes with body mass index variability. PLoS One. 2014;9(5):e96411. doi: 10.1371/journal.pone.0096411 24804925 PMC4013025

[18] Clark MF, Baudouin SV. A systematic review of the quality of genetic association studies in human sepsis. Intensive Care Med. 2006;32(11):1706–12. doi: 10.1007/s00134-006-0327-y 16957907

[19] Minelli C, Thompson JR, Abrams KR, Thakkinstian A, Attia J. The choice of a genetic model in the meta-analysis of molecular association studies. Int J Epidemiol. 2005;34(6):1319–28. doi: 10.1093/ije/dyi169 16115824

[20] Castano-Rodriguez N, Diaz-Gallo LM, Pineda-Tamayo R, Rojas-Villarraga A, Anaya JM. Meta-analysis of HLA-DRB1 and HLA-DQB1 polymorphisms in Latin American patients with systemic lupus erythematosus. Autoimmun Rev. 2008;7(4):322–30. doi: 10.1016/j.autrev.2007.12.002 18295738

[21] Higgins JP, Thompson SG. Quantifying heterogeneity in a meta-analysis. Stat Med. 2002;21(11):1539–58. doi: 10.1002/sim.1186 12111919

[22] Higgins JP, Thompson SG, Deeks JJ, Altman DG. Measuring inconsistency in meta-analyses. BMJ. 2003;327(7414):557–60. doi: 10.1136/bmj.327.7414.557 12958120 PMC192859

[23] Agwa SHA, Kamel MM, Elghazaly H, Abd Elsamee AM, Hafez H, Girgis SA, et al. Association between interferon-lambda-3 rs12979860, tll1 rs17047200 and ddr1 rs4618569 variant polymorphisms with the course and outcome of sars-cov-2 patients. Genes. 2021;12(6). doi: 10.3390/genes12060830 34071309 PMC8230293

[24] Alghamdi J, Alaamery M, Barhoumi T, Rashid M, Alajmi H, Aljasser N, et al. Interferon-induced transmembrane protein-3 genetic variant rs12252 is associated with COVID-19 mortality. Genomics. 2021;113(4):1733–41. doi: 10.1016/j.ygeno.2021.04.002 33838280 PMC8025598

[25] Amodio E, Pipitone RM, Grimaudo S, Immordino P, Maida CM, Prestileo T, et al. SARS-CoV-2 viral load, ifnλ polymorphisms and the course of COVID-19: An observational study. Journal of Clinical Medicine. 2020;9(10):1–9. doi: 10.3390/jcm9103315 33076493 PMC7602550

[26] Amoroso A, Magistroni P, Vespasiano F, Bella A, Bellino S, Puoti F, et al. HLA and AB0 Polymorphisms May Influence SARS-CoV-2 Infection and COVID-19 Severity. Transplantation. 2021;105(1):193–200. doi: 10.1097/TP.0000000000003507 33141807

[27] Avendaño-Félix M, Ochoa-Ramírez LA, Ramos-Payán R, Aguilar-Medina M, Ayala-Ham A, Rendón-Aguilar H, et al. Lack of Effects of the Genetic Polymorphisms of Interleukin-10 in Clinical Outcomes of COVID-19. Viral immunology. 2021. doi: 10.1089/vim.2021.0022 34115949

[28] Benetti E, Tita R, Spiga O, Ciolfi A, Birolo G, Bruselles A, et al. ACE2 gene variants may underlie interindividual variability and susceptibility to COVID-19 in the Italian population. Eur J Hum Genet. 2020;28(11):1602–14. doi: 10.1038/s41431-020-0691-z 32681121 PMC7366459

[29] Benetti E, Giliberti A, Emiliozzi A, Valentino F, Bergantini L, Fallerini C, et al. Clinical and molecular characterization of COVID-19 hospitalized patients. PLoS ONE. 2020;15(11 November). doi: 10.1371/journal.pone.0242534 33206719 PMC7673557

[30] Bernas SN, Baldauf H, Wendler S, Heidenreich F, Lange V, Hofmann JA, et al. CCR5Δ32 mutations do not determine COVID-19 disease course. International Journal of Infectious Diseases. 2021;105:653–5. doi: 10.1016/j.ijid.2021.02.108 33667698 PMC7923852

[31] Cabrera-Marante O, de Frías ER, Pleguezuelo DE, Allende LM, Serrano A, Laguna-Goya R, et al. Perforin gene variant A91V in young patients with severe COVID-19. Haematologica. 2020;105(12):2844–6. doi: 10.3324/haematol.2020.260307 33256384 PMC7716361

[32] Cafiero C, Rosapepe F, Palmirotta R, Re A, Ottaiano MP, Benincasa G, et al. Angiotensin system polymorphisms’ in sars-cov-2 positive patients: Assessment between symptomatic and asymptomatic patients: A pilot study. Pharmacogenomics and Personalized Medicine. 2021;14:621–9. doi: 10.2147/PGPM.S303666 34079337 PMC8166347

[33] Calabrese C, Annunziata A, Coppola A, Pafundi PC, Guarino S, Di Spirito V, et al. ACE Gene I/D Polymorphism and Acute Pulmonary Embolism in COVID19 Pneumonia: A Potential Predisposing Role. Frontiers in Medicine. 2020;7. 33585520 10.3389/fmed.2020.631148 PMC7874110

[34] Cantalupo S, Lasorsa VA, Russo R, Andolfo I, D’alterio G, Rosato BE, et al. Regulatory noncoding and predicted pathogenic coding variants of ccr5 predispose to severe covid-19. International Journal of Molecular Sciences. 2021;22(10). doi: 10.3390/ijms22105372 34065289 PMC8161088

[35] Coto E, Albaiceta GM, Clemente MG, Gómez J. Lack of association between SNPsrs8176719 (O blood group) and COVID-19: Data from Spanish age matched patients and controls. Transfusion. 2021;61(2):654–6. doi: 10.1111/trf.16206 33191530 PMC7753298

[36] Cuesta-Llavona E, Gómez J, Albaiceta GM, Amado-Rodríguez L, García-Clemente M, Gutiérrez-Rodríguez J, et al. Variant-genetic and transcript-expression analysis showed a role for the chemokine-receptor CCR5 in COVID-19 severity. International Immunopharmacology. 2021;98. doi: 10.1016/j.intimp.2021.107825 34116286 PMC8169316

[37] Del Ser T, Fernández-Blázquez MA, Valentí M, Zea-Sevilla MA, Frades B, Alfayate E, et al. Residence, Clinical Features, and Genetic Risk Factors Associated with Symptoms of COVID-19 in a Cohort of Older People in Madrid. Gerontology. 2021. 10.1159/000513182 PMC7900450 33429394

[38] Dite GS, Murphy NM, Allman R. An integrated clinical and genetic model for predicting risk of severe COVID-19: A population-based case-control study. PLoS One. 2021;16(2):e0247205. doi: 10.1371/journal.pone.0247205 33592063 PMC7886160

[39] Ellinghaus D, Degenhardt F, Bujanda L, Buti M, Albillos A, Invernizzi P, et al. Genomewide association study of severe covid-19 with respiratory failure. New England Journal of Medicine. 2020;383(16):1522–34. doi: 10.1056/NEJMoa2020283 32558485 PMC7315890

[40] Gavriilaki E, Asteris PG, Touloumenidou T, Koravou EE, Koutra M, Papayanni PG, et al. Genetic justification of severe COVID-19 using a rigorous algorithm. Clinical Immunology. 2021;226. doi: 10.1016/j.clim.2021.108726 33845193 PMC8043057

[41] Gómez J, Albaiceta GM, García-Clemente M, López-Larrea C, Amado-Rodríguez L, Lopez-Alonso I, et al. Angiotensin-converting enzymes (ACE, ACE2) gene variants and COVID-19 outcome. Gene. 2020;762. doi: 10.1016/j.gene.2020.145102 32882331 PMC7456966

[42] Gómez J, Albaiceta GM, Cuesta-Llavona E, García-Clemente M, López-Larrea C, Amado-Rodríguez L, et al. The Interferon-induced transmembrane protein 3 gene (IFITM3) rs12252 C variant is associated with COVID-19. Cytokine. 2021;137:155354. doi: 10.1016/j.cyto.2020.155354 33113474

[43] Grimaudo S, Amodio E, Pipitone RM, Maida CM, Pizzo S, Prestileo T, et al. PNPLA3 and TLL-1 Polymorphisms as Potential Predictors of Disease Severity in Patients With COVID-19. Frontiers in Cell and Developmental Biology. 2021;9. doi: 10.3389/fcell.2021.627914 34249902 PMC8262646

[44] Gunal O, Sezer O, Ustun GU, Ozturk CE, Sen A, Yigit S, et al. Angiotensin-converting enzyme-1 gene insertion/deletion polymorphism may be associated with COVID-19 clinical severity: a prospective cohort study. Ann Saudi Med. 2021;41(3):141–6. doi: 10.5144/0256-4947.2021.141 34085542 PMC8176375

[45] Hamet P, Pausova Z, Attaoua R, Hishmih C, Haloui M, Shin J, et al. SARS-CoV-2 Receptor ACE2 Gene Is Associated with Hypertension and Severity of COVID 19: Interaction with Sex, Obesity, and Smoking. American journal of hypertension. 2021;34(4):367–76. doi: 10.1093/ajh/hpaa223 33386398 PMC7799248

[46] Hubacek JA, Dlouha L, Dusek L, Majek O, Adamkova V. Apolipoprotein E4 Allele in Subjects with COVID-19. Gerontology. 2021;67(3):320–2. doi: 10.1159/000516200 33965962 PMC8247822

[47] Hubacek JA, Dusek L, Majek O, Adamek V, Cervinkova T, Dlouha D, et al. ACE I/D polymorphism in Czech first-wave SARS-CoV-2-positive survivors. Clinica Chimica Acta. 2021;519:206–9. doi: 10.1016/j.cca.2021.04.024 33957095 PMC8091801

[48] Karakaş Çelik S, Çakmak Genç G, Pişkin N, Açikgöz B, Altinsoy B, Kurucu İşsiz B, et al. Polymorphisms of ACE (I/D) and ACE2 receptor gene (Rs2106809, Rs2285666) are not related to the clinical course of COVID-19: A case study. Journal of Medical Virology. 2021. 10.1002/jmv.27160 PMC8426884 34170561

[49] Kerget F, Kerget B. Frequency of interleukin-6 rs1800795 (-174G/C) and rs1800797 (-597G/A) polymorphisms in COVID-19 patients in Turkey who develop macrophage activation syndrome. Japanese journal of infectious diseases. 2021. 10.7883/yoken.JJID.2021.046 33952771

[50] Kolin DA, Kulm S, Christos PJ, Elemento O. Clinical, regional, and genetic characteristics of Covid-19 patients from UK Biobank. PLoS ONE. 2020;15(11 November). 10.1371/journal.pone.0241264 PMC7671499 33201886

[51] Kuo CL, Pilling LC, Atkins JL, Masoli JAH, Delgado J, Kuchel GA, et al. APOE e4 Genotype Predicts Severe COVID-19 in the UK Biobank Community Cohort. J Gerontol A Biol Sci Med Sci. 2020;75(11):2231–2. doi: 10.1093/gerona/glaa131 32451547 PMC7314139

[52] Latini A, Agolini E, Novelli A, Borgiani P, Giannini R, Gravina P, et al. COVID-19 and Genetic Variants of Protein Involved in the SARS-CoV-2 Entry into the Host Cells. Genes (Basel). 2020;11(9). doi: 10.3390/genes11091010 32867305 PMC7565048

[53] Lehrer S, Rheinstein PH. Homozygosity for rs17775810 Minor Allele Associated With Reduced Mortality of COVID-19 in the UK Biobank Cohort. In Vivo. 2021;35(2):965–8. doi: 10.21873/invivo.12338 33622890 PMC8045077

[54] Lehrer S, Rheinstein PH. ABO blood groups, COVID-19 infection and mortality. Blood Cells Mol Dis. 2021;89:102571. doi: 10.1016/j.bcmd.2021.102571 33894687 PMC8059281

[55] Littera R, Campagna M, Deidda S, Angioni G, Cipri S, Melis M, et al. Human Leukocyte Antigen Complex and Other Immunogenetic and Clinical Factors Influence Susceptibility or Protection to SARS-CoV-2 Infection and Severity of the Disease Course. The Sardinian Experience. Frontiers in Immunology. 2020;11. doi: 10.3389/fimmu.2020.605688 33343579 PMC7746644

[56] Lorente L, Martín MM, Franco A, Barrios Y, Cáceres JJ, Solé-Violán J, et al. HLA genetic polymorphisms and prognosis of patients with COVID-19. Medicina Intensiva. 2020. doi: 10.1016/j.medin.2020.08.004 38620408 PMC7474921

[57] Malaquias MAS, Gadotti AC, Motta-Junior JDS, Martins APC, Azevedo MLV, Benevides APK, et al. The role of the lectin pathway of the complement system in SARS-CoV-2 lung injury. Translational Research. 2020. doi: 10.1016/j.trsl.2020.11.008 33221483 PMC7677075

[58] Martínez-Sanz J, Jiménez D, Martínez-Campelo L, Cruz R, Vizcarra P, Sánchez-Conde M, et al. Role of ACE2 genetic polymorphisms in susceptibility to SARS-CoV-2 among highly exposed but non infected healthcare workers. Emerging Microbes and Infections. 2021;10(1):493–6. doi: 10.1080/22221751.2021.1902755 33704002 PMC7993370

[59] Medetalibeyoglu A, Bahat G, Senkal N, Kose M, Avci K, Sayin GY, et al. Mannose binding lectin gene 2 (rs1800450) missense variant may contribute to development and severity of COVID-19 infection. Infect Genet Evol. 2021;89:104717. doi: 10.1016/j.meegid.2021.104717 33515713 PMC7838598

[60] Möhlendick B, Schönfelder K, Breuckmann K, Elsner C, Babel N, Balfanz P, et al. ACE2 polymorphism and susceptibility for SARS-CoV-2 infection and severity of COVID-19. Pharmacogenetics and genomics. 2021. doi: 10.1097/FPC.0000000000000436 34001841 PMC8415730

[61] Monticelli M, Mele BH, Benetti E, Fallerini C, Baldassarri M, Furini S, et al. Protective role of a tmprss2 variant on severe covid-19 outcome in young males and elderly women. Genes. 2021;12(4). doi: 10.3390/genes12040596 33921689 PMC8073081

[62] Naemi FMA, Al-Adwani S, Al-Khatabi H, Al-Nazawi A. Association between the HLA genotype and the severity of COVID-19 infection among South Asians. J Med Virol. 2021;93(7):4430–7. doi: 10.1002/jmv.27003 33830530 PMC8251353

[63] Novelli A, Biancolella M, Borgiani P, Cocciadiferro D, Colona VL, D’Apice MR, et al. Analysis of ACE2 genetic variants in 131 Italian SARS-CoV-2-positive patients. Hum Genomics. 2020;14(1):29. doi: 10.1186/s40246-020-00279-z 32917283 PMC7483483

[64] Novelli A, Andreani M, Biancolella M, Liberatoscioli L, Passarelli C, Colona VL, et al. HLA allele frequencies and susceptibility to COVID-19 in a group of 99 Italian patients. HLA. 2020;96(5):610–4. doi: 10.1111/tan.14047 32827207 PMC7461491

[65] Pairo-Castineira E, Clohisey S, Klaric L, Bretherick AD, Rawlik K, Pasko D, et al. Genetic mechanisms of critical illness in COVID-19. Nature. 2021;591(7848):92–8. doi: 10.1038/s41586-020-03065-y 33307546

[66] Petrazzuolo A, Le Naour J, Vacchelli E, Gaussem P, Ellouze S, Jourdi G, et al. No impact of cancer and plague-relevant FPR1 polymorphisms on COVID-19. OncoImmunology. 2020;9(1). doi: 10.1080/2162402X.2020.1857112 33344044 PMC7734042

[67] Posadas-Sánchez R, Sánchez-Muñoz F, Guzmán-Martín CA, Hernández-Díaz Couder A, Rojas-Velasco G, Fragoso JM, et al. Dipeptidylpeptidase-4 levels and DPP4 gene polymorphisms in patients with COVID-19. Association with disease and with severity. Life Sci. 2021;276:119410. doi: 10.1016/j.lfs.2021.119410 33774023 PMC7989663

[68] Ravikanth V, Sasikala M, Naveen V, Latha SS, Parsa KVL, Vijayasarathy K, et al. A variant in TMPRSS2 is associated with decreased disease severity in COVID-19. Meta Gene. 2021;29. doi: 10.1016/j.mgene.2021.100930 34075330 PMC8161869

[69] Russo R, Andolfo I, Lasorsa VA, Cantalupo S, Marra R, Frisso G, et al. The TNFRSF13C H159Y Variant Is Associated with Severe COVID-19: A Retrospective Study of 500 Patients from Southern Italy. Genes (Basel). 2021;12(6). doi: 10.3390/genes12060881 34201032 PMC8226789

[70] Saleh A, Sultan A, Elashry MA, Farag A, Mortada MI, Ghannam MA, et al. Association of TNF-α G-308 a Promoter Polymorphism with the Course and Outcome of COVID-19 Patients. Immunological Investigations. 2020. doi: 10.1080/08820139.2020.1851709 33228423 PMC7711738

[71] Salem Hareedy M, Rashad SM, Hetta HF, Hassanien SM, Abdellatif H, Hassanien M. CYP2D6 and CYP3A4 variants influence the risk and outcome of COVID-19 infection among rheumatoid arthritis patients maintained on hydroxychloroquine. Drug Metabolism and Personalized Therapy. 2021;36(2):99–111. doi: 10.1515/dmdi-2020-0164 33770833

[72] Schönfelder K, Breuckmann K, Elsner C, Dittmer U, Fistera D, Herbstreit F, et al. The influence of IFITM3 polymorphisms on susceptibility to SARS-CoV-2 infection and severity of COVID-19. Cytokine. 2021;142:155492. doi: 10.1016/j.cyto.2021.155492 33711707 PMC7936555

[73] Schönfelder K, Breuckmann K, Elsner C, Dittmer U, Fistera D, Herbstreit F, et al. Transmembrane serine protease 2 Polymorphisms and Susceptibility to Severe Acute Respiratory Syndrome Coronavirus Type 2 Infection: A German Case-Control Study. Frontiers in Genetics. 2021;12. doi: 10.3389/fgene.2021.667231 33968142 PMC8097083

[74] Scutt DG, Overall DA. Single nucleotide polymorphisms in key aging pathways, and phenotypic markers of frailty are associated with increased odds of hospital admission with COVID-19. Journal of Infection. 2021. 10.1016/j.jinf.2021.02.006 PMC7873599 33581239

[75] Shikov AE, Barbitoff YA, Glotov AS, Danilova MM, Tonyan ZN, Nasykhova YA, et al. Analysis of the Spectrum of ACE2 Variation Suggests a Possible Influence of Rare and Common Variants on Susceptibility to COVID-19 and Severity of Outcome. Frontiers in Genetics. 2020;11. 33133145 10.3389/fgene.2020.551220 PMC7550667

[76] Shkurnikov M, Nersisyan S, Jankevic T, Galatenko A, Gordeev I, Vechorko V, et al. Association of HLA Class I Genotypes With Severity of Coronavirus Disease-19. Frontiers in Immunology. 2021;12. 10.3389/fimmu.2021.641900 PMC7959787 33732261

[77] Torre-Fuentes L, Matías-Guiu J, Hernández-Lorenzo L, Montero-Escribano P, Pytel V, Porta-Etessam J, et al. ACE2, TMPRSS2, and Furin variants and SARS-CoV-2 infection in Madrid, Spain. Journal of Medical Virology. 2021;93(2):863–9. doi: 10.1002/jmv.26319 32691890 PMC7404937

[78] Valenti L, Griffini S, Lamorte G, Grovetti E, Uceda Renteria SC, Malvestiti F, et al. Chromosome 3 cluster rs11385942 variant links complement activation with severe COVID-19. J Autoimmun. 2021;117:102595. doi: 10.1016/j.jaut.2021.102595 33453462 PMC7796659

[79] Verma S, Abbas M, Verma S, Khan FH, Raza ST, Siddiqi Z, et al. Impact of I/D polymorphism of angiotensin-converting enzyme 1 (ACE1) gene on the severity of COVID-19 patients. Infect Genet Evol. 2021;91:104801. doi: 10.1016/j.meegid.2021.104801 33676010 PMC7929788

[80] Vietzen H, Zoufaly A, Traugott M, Aberle J, Aberle SW, Puchhammer-Stöckl E. Deletion of the NKG2C receptor encoding KLRC2 gene and HLA-E variants are risk factors for severe COVID-19. Genetics in Medicine. 2021. doi: 10.1038/s41436-020-01077-7 33500568 PMC7835668

[81] Wang F, Huang S, Gao R, Zhou Y, Lai C, Li Z, et al. Initial whole-genome sequencing and analysis of the host genetic contribution to COVID-19 severity and susceptibility. Cell Discovery. 2020;6(1). doi: 10.1038/s41421-020-00231-4 33298875 PMC7653987

[82] Wang W, Zhang W, Zhang J, He J, Zhu F. Distribution of HLA allele frequencies in 82 Chinese individuals with coronavirus disease-2019 (COVID-19). HLA. 2020;96(2):194–6. doi: 10.1111/tan.13941 32424945 PMC7276866

[83] Wulandari L, Hamidah B, Pakpahan C, Damayanti NS, Kurniati ND, Adiatmaja CO, et al. Initial study on TMPRSS2 p.Val160Met genetic variant in COVID-19 patients. Hum Genomics. 2021;15(1):29. doi: 10.1186/s40246-021-00330-7 34001248 PMC8127183

[84] Zhang Y, Qin L, Zhao Y, Zhang P, Xu B, Li K, et al. Interferon-Induced Transmembrane Protein 3 Genetic Variant rs12252-C Associated With Disease Severity in Coronavirus Disease 2019. J Infect Dis. 2020;222(1):34–7. doi: 10.1093/infdis/jiaa224 32348495 PMC7197559

[85] Zhou J, Liu C, Sun Y, Huang W, Ye K. Cognitive disorders associated with hospitalization of COVID-19: Results from an observational cohort study. Brain, Behavior, and Immunity. 2020. doi: 10.1016/j.bbi.2020.10.019 33148439 PMC7584518

[86] Wang Q, Zhang Y, Wu L, Niu S, Song C, Zhang Z, et al. Structural and Functional Basis of SARS-CoV-2 Entry by Using Human ACE2. Cell. 2020;181(4):894–904 e9. doi: 10.1016/j.cell.2020.03.045 32275855 PMC7144619

[87] Hoffmann M, Kleine-Weber H, Schroeder S, Kruger N, Herrler T, Erichsen S, et al. SARS-CoV-2 Cell Entry Depends on ACE2 and TMPRSS2 and Is Blocked by a Clinically Proven Protease Inhibitor. Cell. 2020;181(2):271–80 e8. doi: 10.1016/j.cell.2020.02.052 32142651 PMC7102627

[88] Scialo F, Daniele A, Amato F, Pastore L, Matera MG, Cazzola M, et al. ACE2: The Major Cell Entry Receptor for SARS-CoV-2. Lung. 2020;198(6):867–77. doi: 10.1007/s00408-020-00408-4 33170317 PMC7653219

[89] Rysz S, Al-Saadi J, Sjostrom A, Farm M, Campoccia Jalde F, Platten M, et al. COVID-19 pathophysiology may be driven by an imbalance in the renin-angiotensin-aldosterone system. Nat Commun. 2021;12(1):2417. doi: 10.1038/s41467-021-22713-z 33893295 PMC8065208

[90] Hu Y, Liu L, Lu X. Regulation of Angiotensin-Converting Enzyme 2: A Potential Target to Prevent COVID-19? Front Endocrinol (Lausanne). 2021;12:725967. doi: 10.3389/fendo.2021.725967 34745001 PMC8569797

[91] Magrone T, Magrone M, Jirillo E. Focus on Receptors for Coronaviruses with Special Reference to Angiotensin- Converting Enzyme 2 as a Potential Drug Target—A Perspective. Endocr Metab Immune Disord Drug Targets. 2020;20(6):807–11. doi: 10.2174/1871530320666200427112902 32338224

[92] Yamamoto N, Ariumi Y, Nishida N, Yamamoto R, Bauer G, Gojobori T, et al. SARS-CoV-2 infections and COVID-19 mortalities strongly correlate with ACE1 I/D genotype. Gene. 2020;758:144944. doi: 10.1016/j.gene.2020.144944 32628976 PMC7833925

[93] Gemmati D, Bramanti B, Serino ML, Secchiero P, Zauli G, Tisato V. COVID-19 and Individual Genetic Susceptibility/Receptivity: Role of ACE1/ACE2 Genes, Immunity, Inflammation and Coagulation. Might the Double X-chromosome in Females Be Protective against SARS-CoV-2 Compared to the Single X-Chromosome in Males? Int J Mol Sci. 2020;21(10). 10.3390/ijms21103474 PMC7278991 32423094

[94] Gemmati D, Tisato V. Genetic Hypothesis and Pharmacogenetics Side of Renin-Angiotensin-System in COVID-19. Genes (Basel). 2020;11(9). doi: 10.3390/genes11091044 32899439 PMC7563402

[95] Zhong WG, Wang Y, Zhu H, Zhao X. Meta analysis of angiotensin-converting enzyme I/D polymorphism as a risk factor for preeclampsia in Chinese women. Genet Mol Res. 2012;11(3):2268–76. doi: 10.4238/2012.May.21.1 22653650

[96] Delanghe JR, Speeckaert MM, De Buyzere ML. COVID-19 infections are also affected by human ACE1 D/I polymorphism. Clin Chem Lab Med. 2020;58(7):1125–6. doi: 10.1515/cclm-2020-0425 32286246

[97] Delanghe JR, Speeckaert MM, De Buyzere ML. The host’s angiotensin-converting enzyme polymorphism may explain epidemiological findings in COVID-19 infections. Clin Chim Acta. 2020;505:192–3. doi: 10.1016/j.cca.2020.03.031 32220422 PMC7102561

[98] Hatami N, Ahi S, Sadeghinikoo A, Foroughian M, Javdani F, Kalani N, et al. Worldwide ACE (I/D) polymorphism may affect COVID-19 recovery rate: an ecological meta-regression. Endocrine. 2020;68(3):479–84. doi: 10.1007/s12020-020-02381-7 32542429 PMC7294766

[99] Trowsdale J, Knight JC. Major histocompatibility complex genomics and human disease. Annu Rev Genomics Hum Genet. 2013;14:301–23. doi: 10.1146/annurev-genom-091212-153455 23875801 PMC4426292

[100] Dutta M, Dutta P, Medhi S, Borkakoty B, Biswas D. Polymorphism of HLA class I and class II alleles in influenza A(H1N1)pdm09 virus infected population of Assam, Northeast India. J Med Virol. 2018;90(5):854–60. doi: 10.1002/jmv.25018 29315655

[101] Lin M, Tseng HK, Trejaut JA, Lee HL, Loo JH, Chu CC, et al. Association of HLA class I with severe acute respiratory syndrome coronavirus infection. BMC Med Genet. 2003;4:9. doi: 10.1186/1471-2350-4-9 12969506 PMC212558

[102] Dendrou CA, Petersen J, Rossjohn J, Fugger L. HLA variation and disease. Nat Rev Immunol. 2018;18(5):325–39. doi: 10.1038/nri.2017.143 29292391

[103] Deng H, Yan X, Yuan L. Human genetic basis of coronavirus disease 2019. Signal Transduct Target Ther. 2021;6(1):344. doi: 10.1038/s41392-021-00736-8 34545062 PMC8450706

[104] Diamond MS, Farzan M. The broad-spectrum antiviral functions of IFIT and IFITM proteins. Nat Rev Immunol. 2013;13(1):46–57. doi: 10.1038/nri3344 23237964 PMC3773942

[105] Muller JA, Gross R, Conzelmann C, Kruger J, Merle U, Steinhart J, et al. SARS-CoV-2 infects and replicates in cells of the human endocrine and exocrine pancreas. Nat Metab. 2021;3(2):149–65. doi: 10.1038/s42255-021-00347-1 33536639

[106] Zhang Q, Bastard P, Effort CHG, Cobat A, Casanova JL. Human genetic and immunological determinants of critical COVID-19 pneumonia. Nature. 2022;603(7902):587–98. doi: 10.1038/s41586-022-04447-0 35090163 PMC8957595

[107] Asano T, Boisson B, Onodi F, Matuozzo D, Moncada-Velez M, Maglorius Renkilaraj MRL, et al. X-linked recessive TLR7 deficiency in ~1% of men under 60 years old with life-threatening COVID-19. Sci Immunol. 2021;6(62). doi: 10.1126/sciimmunol.abl4348 34413140 PMC8532080

[108] Zhang Q, Bastard P, Liu Z, Le Pen J, Moncada-Velez M, Chen J, et al. Inborn errors of type I IFN immunity in patients with life-threatening COVID-19. Science. 2020;370(6515). 10.1126/science.abd4570 PMC7857407 32972995

[109] Zeng Z, Lan T, Wei Y, Wei X. CCL5/CCR5 axis in human diseases and related treatments. Genes Dis. 2021. doi: 10.1016/j.gendis.2021.08.004 34514075 PMC8423937

[110] Ray PR, Wangzhou A, Ghneim N, Yousuf MS, Paige C, Tavares-Ferreira D, et al. A pharmacological interactome between COVID-19 patient samples and human sensory neurons reveals potential drivers of neurogenic pulmonary dysfunction. Brain Behav Immun. 2020;89:559–68. doi: 10.1016/j.bbi.2020.05.078 32497778 PMC7263237

[111] Alkhatib G, Combadiere C, Broder CC, Feng Y, Kennedy PE, Murphy PM, et al. CC CKR5: a RANTES, MIP-1alpha, MIP-1beta receptor as a fusion cofactor for macrophage-tropic HIV-1. Science. 1996;272(5270):1955–8. doi: 10.1126/science.272.5270.1955 8658171

[112] Gkouskou K, Vasilogiannakopoulou T, Andreakos E, Davanos N, Gazouli M, Sanoudou D, et al. COVID-19 enters the expanding network of apolipoprotein E4-related pathologies. Redox Biol. 2021;41:101938. doi: 10.1016/j.redox.2021.101938 33730676 PMC7943392

[113] Mahley RW. Apolipoprotein E: from cardiovascular disease to neurodegenerative disorders. J Mol Med (Berl). 2016;94(7):739–46. doi: 10.1007/s00109-016-1427-y 27277824 PMC4921111

[114] El-Lebedy D, Raslan HM, Mohammed AM. Apolipoprotein E gene polymorphism and risk of type 2 diabetes and cardiovascular disease. Cardiovasc Diabetol. 2016;15:12. doi: 10.1186/s12933-016-0329-1 26800892 PMC4724147

[115] Inal J. Biological Factors Linking ApoE epsilon4 Variant and Severe COVID-19. Curr Atheroscler Rep. 2020;22(11):70. doi: 10.1007/s11883-020-00896-y 33006059 PMC7529518

