# Supplementary material for: Genetic polymorphisms associated with susceptibility to COVID-19 disease and severity: A systematic review and meta-analysis
Source: PLoS One. 2022 Jul 6;17(7):e0270627. doi: 10.1371/journal.pone.0270627 (PMC9258831; doi:10.1371/journal.pone.0270627)
Supplement: S1 Table — (DOCX) [file pone.0270627.s001.docx]

**Supplementary Table 1** - Clark-Baudouin quality assessment scale for the studies included in the systematic-review.

| **Author** | **Year** | **Clark-Baudouin Score** |
| --- | --- | --- |
| Agha, SHA. | 2021 | 7 |
| Alghamdi, J. | 2021 | 7 |
| Amodio, E. | 2020 | 6 |
| Amoroso, A. | 2021 | 4 |
| Avendaño-Felix, M. | 2021 | 5 |
| Benetti, E.^a^ | 2020 | 6 |
| Benetti, E.^b^ | 2020 | 6 |
| Bernas, SN. | 2021 | 5 |
| Cabrera-Marante, O. | 2020 | 2 |
| Cafiero, C. | 2021 | 6 |
| Calabrese, C. | 2021 | 4 |
| Coto, E. | 2020 | 6 |
| Cuesta-Llavona, E. | 2021 | 7 |
| Del Ser, T. | 2021 | 5 |
| Dite, GS. | 2021 | 7 |
| Ellinghaus, D. | 2020 | 7 |
| Gavriilaki, E. | 2021 | 6 |
| Gomez, J | 2021 | 7 |
| Gomez, J. | 2020 | 7 |
| Grimaudo, S. | 2021 | 8 |
| Gunal, O. | 2021 | 7 |
| Hamet, P. | 2021 | 8 |
| Hu, J. | 2021 | 5 |
| Hubacek, JA^c^ | 2021 | 6 |
| Hubacek, JA^b^ | 2021 | 4 |
| Hubacek, JA.^a^ | 2021 | 5 |
| Karakas Çelik, S. | 2021 | 5 |
| Kerget, F. | 2021 | 5 |
| Kolin, DA. | 2020 | 7 |
| Kuo, CL. | 2020 | 4 |
| Latini, A | 2020 | 8 |
| Lehrer, S.^a^ | 2021 | 3 |
| Lehrer, S.^b^ | 2021 | 5 |
| Littera, R. | 2020 | 6 |
| Lorente, L | 2020 | 5 |
| Malaquias, M. | 2020 | 5 |
| Martinez Sanz, J | 2021 | 6 |
| Medetalibeyoglu, A. | 2021 | 7 |
| Mohlendick, B. | 2021 | 7 |
| Monticelli, M. | 2021 | 6 |
| Naemi, FMA. | 2021 | 7 |
| Novelli,A^a^ | 2020 | 6 |
| Novelli,A^b^ | 2020 | 6 |
| Pairo-Castineira, E. | 2020 | 8 |
| Petrazzuolo, A. | 2020 | 6 |
| Posadas-Sanchez, R. | 2021 | 7 |
| Ravikanth, V. | 2021 | 7 |
| Russo, R. | 2021 | 6 |
| Saleh, A | 2020 | 5 |
| Salem Hareedy, M. | 2021 | 7 |
| Schonfelder, K.^a^ | 2021 | 6 |
| Schonfelder, K.^b^ | 2021 | 7 |
| Scutt, G | 2021 | 7 |
| Shikov, AE. | 2020 | 7 |
| Shkurnikov, M. | 2021 | 5 |
| Torre-Fuentes, L. | 2020 | 5 |
| Valenti, L. | 2021 | 6 |
| Verma, S. | 2021 | 6 |
| Vietzen, H. | 2021 | 7 |
| Wang, F. | 2020 | 7 |
| Wang, W. | 2020 | 6 |
| Wulandari, L. | 2021 | 6 |
| Zhang, Y. | 2020 | 7 |
| Zhou, J. | 2021 | 6 |

The CBS consists in a scoring system that allows from 0 to 10 points regarding pre-defined criteria. Most of the included studies were classified as presenting good quality since 70.1 % of the studies were awarded 6 to 8 points; 29.1% of the articles were awarded with <6 points.
