# Supplementary material for: Genetic polymorphisms associated with susceptibility to COVID-19 disease and severity: A systematic review and meta-analysis
Source: PLoS One. 2022 Jul 6;17(7):e0270627. doi: 10.1371/journal.pone.0270627 (PMC9258831; doi:10.1371/journal.pone.0270627)
Supplement: S3 Table — (DOCX) [file pone.0270627.s003.docx]

**Supplementary Table 3.** Meta-analyses of the association between polymorphisms in HLA and COVID-19.

| **HLA** | **Studies** | **I^2^** | **Model** | **OR (95% CI)** |
| --- | --- | --- | --- | --- |
| HLA-A*01 | 3 | 27.2% | Fixed | 0.96 (0.81 – 1.15) |
| HLA-A*02 | 3 | 3.7% | Fixed | 0.96 (0.85 – 1.09) |
| HLA-A*03 | 3 | 37.9% | Fixed | 1.14 (0.96 – 1.35) |
| HLA-A*11 | 3 | 52.7% | Random | 1.12 (0.77 – 1.62) |
| HLA-A*23 | 3 | 61.5% | Random | 0.82 (0.42 – 1.60) |
| HLA-A*24 | 3 | 71.0% | Random | 1.08 (0.70 – 1.67) |
| HLA-A*25 | 3 | 0.0% | Fixed | 1.26 (0.79 – 2.01) |
| HLA-A*26 | 3 | 43.4% | Fixed | 1.01 (0.78 – 1.31) |
| HLA-A*29 | 3 | 0.0% | Fixed | 0.75 (0.55 – 1.03) |
| HLA-A*30 | 3 | 0.0% | Fixed | 0.79 (0.64 – 0.98)* |
| HLA-A*31 | 3 | 79.2% | Random | 0.79 (0.26 – 2.44) |
| HLA-A*32 | 3 | 72.3% | Random | 0.83 (0.46 – 1.51) |
| HLA-A*33 | 3 | 0.0% | Fixed | 0.76 (0.54 – 1.08) |
| HLA-A*68 | 3 | 0.0% | Fixed | 1.09 (0.81 – 1.47) |
| HLA-B*07 | 3 | 8.4% | Fixed | 1.00 (0.79 – 1.26) |
| HLA-B*08 | 3 | 26.7% | Fixed | 0.97 (0.75 – 1.25) |
| HLA-B*13 | 3 | 0.0% | Fixed | 1.36 (0.97 – 1.89) |
| HLA-B*14 | 3 | 27.3% | Fixed | 0.96 (0.74 – 1.26) |
| HLA-B*15 | 3 | 0.0% | Fixed | 0.94 (0.73 – 1.21) |
| HLA-B*18 | 3 | 65.8% | Random | 1.03 (0.74 – 1.44) |
| HLA-B*27 | 3 | 0.0% | Fixed | 1.13 (0.76 – 1.66) |
| HLA-B*35 | 3 | 0.0% | Fixed | 1.12 (0.96 – 1.31) |
| HLA-B*37 | 3 | 0.0% | Fixed | 1.25 (0.78 – 1.98) |
| HLA-B*38 | 3 | 0.0% | Fixed | 0.93 (0.66 – 1.30) |
| HLA-B*39 | 3 | 67.4% | Random | 1.38 (0.71 – 2.70) |
| HLA-B*40 | 3 | 76.0% | Random | 1.46 (0.64 – 3.33) |
| HLA-B*41 | 3 | 0.0% | Fixed | 0.85 (0.52 – 1.38) |
| HLA-B*44 | 3 | 59.0% | Random | 1.10 (0.77 – 1.56) |
| HLA-B*45 | 3 | 52.9% | Random | 1.14 (0.47 – 2.77) |
| HLA-B*47 | 3 | 0.0% | Fixed | 0.84 (0.37 – 1.88) |
| HLA-B*49 | 3 | 0.0% | Fixed | 0.85 (0.64 – 1.13) |
| HLA-B*50 | 3 | 0.0% | Fixed | 0.85 (0.58 – 1.24) |
| HLA-B*51 | 3 | 51.7% | Random | 1.15 (0.85 – 1.55) |
| HLA-B*52 | 3 | 0.0% | Fixed | 0.85 (0.54 – 1.32) |
| HLA-B*53 | 3 | 80.1% | Random | 1.38 (0.35 – 5.38) |
| HLA-B*55 | 3 | 73.2% | Random | 0.42 (0.09 – 1.97) |
| HLA-B*57 | 3 | 0.0% | Fixed | 0.85 (0.60 – 1.19) |
| HLA-B*58 | 3 | 73.9% | Random | 0.84 (0.40 – 1.77) |
| HLA-B*58:01 | 3 | 48.3% | Fixed | 1.38 (0.93 – 2.05) |
| HLA-C*02 | 3 | 0.0% | Fixed | 1.06 (0.83 – 1.36) |
| HLA-C*03 | 3 | 14.4% | Fixed | 0.86 (0.67 – 1.10) |
| HLA-C*04 | 3 | 80.7% | Random | 1.18 (0.78 – 1.79) |
| HLA-C*05 | 3 | 0.0% | Fixed | 0.87 (0.71 – 1.07) |
| HLA-C*06 | 3 | 0.0% | Fixed | 0.96 (0.79 – 1.16) |
| HLA-C*06:02 | 3 | 72.6% | Random | 0.74 (0.38 – 1.43) |
| HLA-C*07 | 3 | 36.1% | Fixed | 0.91 (0.81 – 1.04) |
| HLA-C*08 | 3 | 20.9% | Fixed | 0.92 (0.70 – 1.20) |
| HLA-C*12 | 3 | 55.2% | Random | 1.19 (0.85 – 1.67) |
| HLA-C*14 | 3 | 4.9% | Fixed | 1.04 (0.70 – 1.54) |
| HLA-C*15 | 3 | 61.7% | Random | 0.97 (0.54 – 1.74) |
| HLA-C*16 | 3 | 70.6% | Random | 1.46 (0.88 – 2.40) |
| HLA-C*17 | 3 | 0.0% | Fixed | 0.89 (0.54 – 1.46) |
| HLA-DRB1*01 | 3 | 0.0% | Fixed | 1.13 (0.94 – 1.36) |
| HLA-DRB1*03 | 3 | 10.2% | Fixed | 0.89 (0.75 – 1.06) |
| HLA-DRB1*04 | 3 | 27.2% | Fixed | 0.87 (0.72 – 1.04) |
| HLA-DRB1*07 | 4 | 33.5% | Fixed | 0.89 (0.76 – 1.04) |
| HLA-DRB1*08 | 4 | 53.7% | Random | 1.35 (0.88 – 2.09) |
| HLA-DRB1*10 | 3 | 0.0% | Fixed | 1.09 (0.70 – 1.69) |
| HLA-DRB1*11 | 3 | 49.0% | Fixed | 1.01 (0.88 – 1.16) |
| HLA-DRB1*12 | 3 | 46.3% | Fixed | 1.06 (0.83 – 1.60) |
| HLA-DRB1*13 | 3 | 0.0% | Fixed | 1.02 (0.85 – 1.21) |
| HLA-DRB1*14 | 3 | 54.8% | Random | 1.32 (0.79 – 2.20) |
| HLA-DRB1*15 | 3 | 0.0% | Fixed | 0.91 (0.73 – 1.13) |
| HLA-DRB1*16 | 3 | 0.0% | Fixed | 1.12 (0.92 – 1.36) |

OR: odds ratio; CI: confidence interval. * Indicates a significant association at P < 0.05.
