# Supplementary material for: Genetic polymorphisms associated with susceptibility to COVID-19 disease and severity: A systematic review and meta-analysis
Source: PLoS One. 2022 Jul 6;17(7):e0270627. doi: 10.1371/journal.pone.0270627 (PMC9258831; doi:10.1371/journal.pone.0270627)
Supplement: S4 Table — (DOCX) [file pone.0270627.s004.docx]

**Supplementary Table 4.** Meta-analyses of the association between polymorphisms in *HLA* and COVID-19 severity.

| **HLA** | **Studies** | **I^2^** | **Model** | **OR (95% CI)** |
| --- | --- | --- | --- | --- |
| HLA-A*01 | 5 | 29.7% | Fixed | 1.23 (0.96 – 1.57) |
| HLA-A*02 | 5 | 0.0% | Fixed | 0.01 (0.86 – 1.20) |
| HLA-A*03 | 5 | 0.0% | Fixed | 1.06 (0.84 – 1.33) |
| HLA-A*11 | 5 | 54.3% | Random | 1.33 (0.82 – 2.15) |
| HLA-A*23 | 5 | 40.4% | Fixed | 1.28 (0.81 – 2.03) |
| HLA-A*24 | 5 | 8.4% | Fixed | 0.87 (0.68 – 1.10) |
| HLA-A*25 | 4 | 0.0% | Fixed | 1.20 (0.69 – 2.10) |
| HLA-A*26 | 5 | 0.0% | Fixed | 1.01 (0.72 – 1.43) |
| HLA-A*29 | 5 | 0.0% | Fixed | 1.01 (0.74 – 1.36) |
| HLA-A*30 | 5 | 0.0% | Fixed | 0.86 (0.62 – 1.18) |
| HLA-A*31 | 5 | 0.0% | Fixed | 0.88 (0.53 – 1.45) |
| HLA-A*32 | 5 | 0.0% | Fixed | 0.79 (0.55 – 1.12) |
| HLA-A*33 | 5 | 0.0% | Fixed | 0.56 (0.36 – 0.88)* |
| HLA-A*68 | 5 | 34.2% | Fixed | 0.98 (0.66 – 1.45) |
| HLA-B*07 | 5 | 63.9% | Random | 0.97 (0.54 – 1.76) |
| HLA-B*08 | 5 | 0.0% | Fixed | 0.85 (0.61 – 1.17) |
| HLA-B*13 | 5 | 0.0% | Fixed | 1.59 (0.94 – 2.68) |
| HLA-B*14 | 5 | 33.6% | Fixed | 0.92 (0.66 – 1.29) |
| HLA-B*15 | 5 | 27.3% | Fixed | 1.03 (0.75 – 1.40) |
| HLA-B*18 | 4 | 0.0% | Fixed | 1.10 (0.86 – 1.40) |
| HLA-B*27 | 5 | 0.0% | Fixed | 1.01 (0.63 – 1.62) |
| HLA-B*35 | 5 | 54.5% | Random | 0.91 (0.62 – 1.34) |
| HLA-B*37 | 5 | 0.0% | Fixed | 1.55 (0.84 – 2.87) |
| HLA-B*38 | 5 | 0.0% | Fixed | 1.64 (1.03 – 2.60)* |
| HLA-B*39 | 4 | 0.0% | Fixed | 0.83 (0.49 – 1.39) |
| HLA-B*40 | 5 | 59.1% | Random | 1.15 (0.63 – 2.11) |
| HLA-B*41 | 4 | 0.0% | Fixed | 0.57 (0.28 – 1.16) |
| HLA-B*44 | 5 | 0.0% | Fixed | 0.97 (0.78 – 1.21) |
| HLA-B*45 | 4 | 0.0% | Fixed | 1.28 (0.55 – 2.94) |
| HLA-B*49 | 5 | 0.0% | Fixed | 0.97 (0.65 – 1.44) |
| HLA-B*50 | 4 | 0.6% | Fixed | 0.80 (0.47 – 1.36) |
| HLA-B*51 | 5 | 12.9% | Fixed | 1.08 (0.86 – 1.37) |
| HLA-B*52 | 5 | 28.6% | Fixed | 1.13 (0.65 – 1.97) |
| HLA-B*53 | 4 | 58.9% | Random | 0.84 (0.24 – 2.96) |
| HLA-B*55 | 4 | 0.0% | Fixed | 0.91 (0.50 – 1.67) |
| HLA-B*57 | 5 | 0.0% | Fixed | 1.19 (0.75 – 1.89) |
| HLA-B*58 | 5 | 51.8% | Random | 0.91 (0.43 – 1.94) |
| HLA-C*02 | 5 | 0.0% | Fixed | 0.97 (0.68 – 1.38) |
| HLA-C*03 | 5 | 40.9% | Fixed | 0.96 (0.72 – 1.28) |
| HLA-C*04 | 5 | 59.4% | Random | 0.91 (0.61 – 1.36) |
| HLA-C*05 | 4 | 0.0% | Fixed | 0.89 (0.69 – 1.16) |
| HLA-C*06 | 5 | 0.2% | Fixed | 1.31 (1.00 – 1.72)* |
| HLA-C*07 | 5 | 0.0% | Fixed | 1.04 (0.88 – 1.23) |
| HLA-C*08 | 5 | 43.2% | Fixed | 0.89 (0.64 – 1.23) |
| HLA-C*12 | 5 | 60.3% | Random | 0.96 (0.57 – 1.62) |
| HLA-C*14 | 4 | 0.0% | Fixed | 1.54 (0.90 – 2.61) |
| HLA-C*15 | 5 | 0.0% | Fixed | 0.91 (0.66 – 1.26) |
| HLA-C*16 | 5 | 26.4% | Fixed | 0.95 (0.72 – 1.26) |
| HLA-C*17 | 4 | 0.0% | Fixed | 0.70 (0.34 – 1.42) |
| HLA-DRB1*01 | 5 | 0.0% | Fixed | 0.87 (0.69 – 1.09) |
| HLA-DRB1*03 | 5 | 0.0% | Fixed | 0.92 (0.74 – 1.14) |
| HLA-DRB1*04 | 5 | 0.0% | Fixed | 1.25 (0.98 – 1.60) |
| HLA-DRB1*07 | 5 | 0.0% | Fixed | 0.98 (0.80 – 1.21) |
| HLA-DRB1*08 | 5 | 44.0% | Fixed | 1.34 (0.86 – 2.08) |
| HLA-DRB1*10 | 5 | 0.0% | Fixed | 1.05 (0.61 – 1.81) |
| HLA-DRB1*11 | 5 | 62.5% | Random | 0.79 (0.49 – 1.28) |
| HLA-DRB1*12 | 4 | 41.1% | Fixed | 1.23 (0.65 – 2.31) |
| HLA-DRB1*13 | 5 | 6.6% | Fixed | 1.09 (0.86 – 1.36) |
| HLA-DRB1*14 | 5 | 0.0% | Fixed | 1.04 (0.73 – 1.49) |
| HLA-DRB1*15 | 5 | 0.0% | Fixed | 0.84 (0.66 – 1.07) |
| HLA-DRB1*16 | 5 | 0.0% | Fixed | 1.06 (0.76 – 1.49) |
| HLA-DQB1*02 | 4 | 0.0% | Fixed | 0.94 (0.79 – 1.12) |
| HLA-DQB1*03 | 3 | 0.0% | Fixed | 1.18 (0.99 – 1.39) |
| HLA-DQB1*04 | 4 | 20.8% | Fixed | 1.24 (0.77 – 1.99) |
| HLA-DQB1*05 | 4 | 41.3% | Fixed | 1.00 (0.83 – 1.21) |
| HLA-DQB1*06 | 4 | 0.0% | Fixed | 0.89 (0.74 – 1.08) |
| HLA-DQA1*01 | 4 | 55.5% | Random | 1.03 (0.81 – 1.31) |
| HLA-DQA1*02 | 3 | 0.0% | Fixed | 0.98 (0.79 – 1.21) |
| HLA-DQA1*03 | 4 | 20.5% | Fixed | 1.00 (0.79 – 1.26) |
| HLA-DQA1*04 | 4 | 0.0% | Fixed | 1.29 (0.79 – 2.12) |
| HLA-DQA1*05 | 4 | 60.6% | Random | 0.89 (0.65 – 1.22) |

OR: odds ratio; CI: confidence interval. * Indicates a significant association at P < 0.05.
