# Supplementary material for: Genetic polymorphisms associated with susceptibility to COVID-19 disease and severity: A systematic review and meta-analysis
Source: PLoS One. 2022 Jul 6;17(7):e0270627. doi: 10.1371/journal.pone.0270627 (PMC9258831; doi:10.1371/journal.pone.0270627)
Supplement: Attachment — Submitted filename: Response to Reviewers.docx [file pone.0270627.s005.docx]

April, 22 2022

To

Giuseppe Novelli

*Academic Editor, PLOS ONE*

Dear Editor:

Thank you for your letter concerning our manuscript.

We appreciated the Reviewer's comments, which helped us to further improve the manuscript. In the following pages you will find our answers for each of the Reviewer's comments. We hope that you will find these answers and the revised version of the manuscript satisfactory. All modifications to the original manuscript are indicated by red fountain.

We look forward to receive your feedback on this revised manuscript.

Sincerely yours,

Profª. Drª. Daisy Crispim

**Point-by-point answers to the Editor comments:**

*A comprehensive study should include some of the most notable findings from the past two years in this field. Indeed, several clinical and immunological studies have shown that type I interferons (IFN-I) play critical roles in the control and pathogenesis of COVID-19. This notion is supported by extensive sequencing of numerous patients with severe COVID-19 who have identified pathogenic mutations in genes that code for active proteins in the interferon circuit. The characterization of autoantibodies capable of neutralizing IFN-I in 10-15% of severe patients allows us to state that COVID-19 can be defined as an interferonopathy. This must be included in Discussion and the references below must be cited:*

*Zhang Q, Bastard P, Effort CHG, Cobat A, Casanova JL. Human genetic and immunological determinants of critical COVID-19 pneumonia. Nature. 2022.* [*https://doi.org/10.1038/s41586-022-04447-0*](https://doi.org/10.1038/s41586-022-04447-0)*. Epub ahead of print.
Asano T, Boisson B, Onodi F, Matuozzo D, Moncada-Velez M, Maglorius Renkilaraj MRL, et al. X-linked recessive TLR7 deficiency in ~1% of men under 60 years old with life-threatening COVID-19. Sci Immunol. 2021 Aug 19;6(62):eabl4348.* [*https://doi.org/10.1126/sciimmunol.abl4348*](https://doi.org/10.1126/sciimmunol.abl4348) *Zhang Q, Bastard P, Liu Z, Le Pen J, Moncada-Velez M, Chen J, et al. Inborn errors of type I IFN immunity in patients with life-threatening COVID-19. Science. 2020 Oct 23;370(6515):eabd4570.* [*https://doi.org/10.1126/science.abd4570*](https://doi.org/10.1126/science.abd4570)*. Epub 2020 Sep 24.*

**Answer:** Thank you for the suggestions. We included a discussion about this topic on the Discussion Section (page 34).

**Point-by-point answers to the Reviewer’s comments:**

**Reviewer #1:**

*The manuscript describes a systematic review of the literature regarding the possible involvement of genetic factors in the susceptibility to SARS-CoV-2 infection.

The main points are consistent with the analysis carried out, according to the Preferred Reporting Items for Systematic Review and Meta-analysis statement (PRISMA). The inclusion and exclusion criteria are well reported. The authors provide a needed synopsis on the current status of the topic, shedding light on the limitations and future perspectives that might be useful for future analysis. The structure of the review is clear and well organised.
The article is interesting and well-focused in the Methods, Results and Discussion parts.*

**Answer:** Thank you for your comments.

*I have only few comments:*

*Table 1 shows all the studies included in the systematic review. They would be easier to consult if they were split between investigating genetic factors that may influence COVID-19 susceptibility and those involved in severity.
Moreover, P-value and OR are should be reported, where possible, for all included studies.*
**Answer:** Thank you for your suggestion. We now included the P-values and ORs, when available, for all included studies. Since many studies reported both results for COVID-19 susceptibility and severity, we opted to not split the table according to results. However, we re-organized our table to make easy to identify those studies associated with susceptibility, severity or both, as you can see in the example below.

| **Reference** | **Population** | **Sample**  **(case/control)** | **Gene** | **Results** |
| --- | --- | --- | --- | --- |
| Agwa *et al*, 2021 | Egyptian | 141 cases /  100 controls | *INFλ*, *TLL1*,  *DDR1* | Disease susceptibility: The *IFN-λ* rs12979860 C/C, *TLL1* rs17047200 A/A and the *DDR1* rs4618569 A/A genotypes were associated with COVID-19 (P = 0.011; P = 0.012 and P = 0.026, respectively).  Severity: The *DDR1* rs4618569 A/G was associated with COVID-19 severity (P = 0.007). |

*The location of the polymorphisms included in meta-analyses should be indicate.*

**Answer:** Thank you for your suggestion. We added the information about the location of the polymorphisms included in meta-analyses on **Table 2** (page 24).

*The title of the paragraph " Search strategy and eligibility criteria" could also be “Literature Search strategy and eligibility criteria”*
**Answer:** We have changed the title according to your suggestion (page 4).

*I suggest to further discuss the issue of genetic variability among different populations, since the majority of studies were conducted in different ethnic groups.*

**Answer:** Thank you for the suggestion. We now included a discussion about genetic variability on the limitation paragraph of the Discussion Section (page 37).

**Reviewer #2:**

*In this manuscript the authors provide a systematic review and meta-analysis of current literature, investigating the association of polymorphisms with COVID-19 susceptibility and severity. The objectives of the analysis are clearly stated and the informations on the search are provided (sources, used terms for literature search). Inclusion and exclusion criteria are clearly stated. Characteristics of the selected studies are complete and well resumed in Table 1 and S2 table. Statistical methods seems appropriate and results are well displayed. I particularly appreciate that the authors have correctly probed the limitations of this study, which can’t be ignored when interpreting the results.*

**Answer:** Thank you for your comment.

*In the manuscript, there are only few minor flaws to be addressed:
1) the title should emphasize the analyses conducted on the association between genetic factors and COVID-19 severity too, since in the discussion it has an equal relevance compared to analyses on the association with COVID-19 susceptibility;*

**Answer:** Thank you for your suggestion. We now also emphasized on the title the association with COVID-19 severity.

*2) reference for the Clark-Baudouin Score (“Data extraction and quality evaluation” section) should be checked, since it doesn’t seem correct;*

**Answer:** We have now included the correct reference (page 6).

*3) I suggest to rephrase the statement “Different comorbidities are associated with a worse COVID-19 outcome, and dementia was among the common comorbidities linked with higher mortality” in the discussion section, since it could convey a message not yet fully supported by scientific evidence, although I can see that is not in the authors’ intentions. As far as I know, there are no studies that have been able to significantly discriminate the contribution of different factors that may underlie an increased risk of COVID-19 mortality in patients with dementia. Due to the characteristic of the pathology, it is not possible not to recognize the relevance of socioeconomic and behavioral factors (failure to observe preventive measures or adherence to therapy). It must also be taken into account that some conditions predisposing to dementia are also risk factors for adverse COVID-19 outcomes (cardiovascular diseases, type 2 diabetes, obesity, asthma, chronic kidney disease). So there are still deeper investigations to be done before dementia itself can be listed as a risk factor associated with higher COVID-19 mortality. Moreover, the cited paper for this statement stresses the focus on the neurological complications of a SARS-CoV-2 infection;*

**Answer:** As suggested, we rephrased this paragraph (Discussion Section, page 35).

*4) unfortunately, it’s not possible to state that this is the first meta-analysis on the field (PMID:34997794), but in my opinion this doesn’t affect the validity of this work, since it is not focused only on the genetic polymorphisms in genes related to the renin-angiotensin-aldosterone system (RAAS), as the cited one.*
**Answer:** Thank you for the information. We excluded this sentence from the Introduction Section.

*In conclusion, for what is in my competence, the manuscript seems carefully conceived and well written. This work, on its current form, provides a promising starting point and it can have an impact in terms of designing broader and deeper investigations.*

**Answer:** Thank you for your comment.

**Journal Requirements:**

*When submitting your revision, we need you to address these additional requirements.*

*1. Please ensure that your manuscript meets PLOS ONE's style requirements, including those for file naming. The PLOS ONE style templates can be found at* 
[*https://journals.plos.org/plosone/s/file?id=wjVg/PLOSOne_formatting_sample_main_body.pdf*](https://journals.plos.org/plosone/s/file?id=wjVg/PLOSOne_formatting_sample_main_body.pdf) *and* 
[*https://journals.plos.org/plosone/s/file?id=ba62/PLOSOne_formatting_sample_title_authors_affiliations.pdf*](https://journals.plos.org/plosone/s/file?id=ba62/PLOSOne_formatting_sample_title_authors_affiliations.pdf)

**Answer:** Our manuscript meets PLOS ONE's style requirements, including those for file naming.

*2. Thank you for stating the following in the Acknowledgments Section of your manuscript:*

*"This study was partially supported by grants from the Conselho Nacional de
Desenvolvimento Científico e Tecnológico (CNPq, grant number 401610/2020-9),
Fundo de Incentivo à Pesquisa e Eventos (FIPE) at Hospital de Clínicas de Porto Alegre
(grant number: 2020-0218), and Coordenação de Aperfeiçoamento de Pessoal de Nível
Superior (CAPES). D.C., C.B.L. and N.E.L are recepients of a scholarship from CNPq,
while C.D. is a recipient of scholarship from CAPES. "*

*We note that you have provided funding information that is not currently declared in your Funding Statement. However, funding information should not appear in the Acknowledgments section or other areas of your manuscript. We will only publish funding information present in the Funding Statement section of the online submission form. 
Please remove any funding-related text from the manuscript and let us know how you would like to update your Funding Statement. Currently, your Funding Statement reads as follows:*

*"This study was partially supported by grants from the Conselho Nacional de Desenvolvimento Científico e Tecnológico (CNPq, grant number 401610/2020-9), Fundo de Incentivo à Pesquisa e Eventos (FIPE) at Hospital de Clínicas de Porto Alegre (grant number: 2020-0218), and Coordenação de Aperfeiçoamento de Pessoal de Nível Superior (CAPES). D.C., C.B.L. and N.E.L are recepients  of a scholarship from CNPq, while C.D. is a recipient of scholarship from CAPES. "*

*Please include your amended statements within your cover letter; we will change the online submission form on your behalf.*

**Answer:** We removed the funding information from our manuscript. The corrected funding information is:

This study was partially supported by grants from the Conselho Nacional de Desenvolvimento Científico e Tecnológico (CNPq, grant numbers 401610/2020-9 and 425579/2018-2), Fundo de Incentivo à Pesquisa e Eventos (FIPE) at Hospital de Clínicas de Porto Alegre (grant number: 2020-0218), and Coordenação de Aperfeiçoamento de Pessoal de Nível Superior (CAPES). D.C., C.B.L. and N.E.L are recipients  of a scholarship from CNPq, while C.D. is a recipient of scholarship from CAPES.

*3. We note that you have included the phrase “data not shown” in your manuscript. Unfortunately, this does not meet our data sharing requirements. PLOS does not permit references to inaccessible data. We require that authors provide all relevant data within the paper, Supporting Information files, or in an acceptable, public repository. Please add a citation to support this phrase or upload the data that corresponds with these findings to a stable repository (such as Figshare or Dryad) and provide and URLs, DOIs, or accession numbers that may be used to access these data. Or, if the data are not a core part of the research being presented in your study, we ask that you remove the phrase that refers to these data.*

**Answer:** We removed the phrase “data not shown” and added the results for the referred sentence (page 28).

*4.  Please review your reference list to ensure that it is complete and correct. If you have cited papers that have been retracted, please include the rationale for doing so in the manuscript text, or remove these references and replace them with relevant current references. Any changes to the reference list should be mentioned in the rebuttal letter that accompanies your revised manuscript. If you need to cite a retracted article, indicate the article’s retracted status in the References list and also include a citation and full reference for the retraction notice.*

**Answer:** Thank you for the comment. We have revised the reference list to ensure that is correct. We did not cite a retracted paper in the present manuscript. We cited 3 articles that have an erratum publication; however, it did not interfere with the results or interpretation of our article.

- Zhou F, Yu T, Du R, Fan G, Liu Y, Liu Z, et al. Clinical course and risk factors for mortality of adult inpatients with COVID-19 in Wuhan, China: a retrospective cohort study. Lancet. 2020;395(10229):1054-62

Erratum: In this Article, the units for d-dimer, haemoglobin, and high-sensitivity cardiac troponin I have been corrected to μg/mL (d-dimer), g/L (haemoglobin), and pg/mL (high-sensitivity cardiac troponin I). In figure 1, the start of systematic corticosteroid for non-survivors has been changed to day 13 after illness onset.

- Valenti L, Griffini S, Lamorte G, Grovetti E, Uceda Renteria SC, Malvestiti F, et al. Chromosome 3 cluster rs11385942 variant links complement activation with severe COVID-19. J Autoimmun. 2021;117:102595

Erratum: Although the correct term of the variant is “rs11385942” it incorrectly appears as “rs11385949”. In table 1, the values of “Cardiometabolic comorbidities” of patients with the G/G variant (2nd column, line 18) and those of patients with the G/GA variant (3rd column, line 18) were reversed and should be: G/G = 1.3 ± 1.0 and G/GA = 0.7 ± 0.7.

- El-Lebedy D, Raslan HM, Mohammed AM. Apolipoprotein E gene polymorphism and risk of type 2 diabetes and cardiovascular disease. Cardiovasc Diabetol. 2016;15:12.

Erratum: In Table 1 provided in the accepted submission, “13.00 ± 6.97” had mistakenly been included below the Diabetes duration row in the T2DM + CVD column.
